# GlioSurv: interpretable transformer for multimodal, individualized survival prediction in diffuse glioma

**DOI:** 10.1038/s41746-025-02018-x

**Published:** 2025-11-14

**Authors:** Junhyeok Lee, Joon Jang, Heeseong Eum, Han Jang, Minchul Kim, Sung Hye Park, Chul Kee Park, Seung Hong Choi, Sung Soo Ahn, Yoseob Han, Kyu Sung Choi

**Affiliations:** 1 Interdisciplinary Programs in Cancer Biology, Seoul National University Graduate School, Seoul, Republic of Korea; 2 Department of Biomedical Sciences, Seoul National University, Seoul, Republic of Korea; 3 Department of Radiology, Seoul National University Hospital, Seoul, Republic of Korea; 4 Department of Radiology, Kangbuk Samsung Hospital, Sungkyunkwan University School of Medicine, Seoul, Republic of Korea; 5 Department of Pathology, Seoul National University Hospital, Seoul, Republic of Korea; 6 Department of Neurosurgery, Seoul National University Hospital, Seoul, Republic of Korea; 7 Department of Radiology, Research Institute of Radiological Science, Center for Clinical Imaging Data Science, Yonsei University College of Medicine, Seoul, Republic of Korea; 8 Department of Electronic Engineering, Soongsil University, Seoul, Republic of Korea; 9 Department of Intelligent Semiconductors, Soongsil University, Seoul, Republic of Korea; 10 Department of Radiology, Seoul National University College of Medicine, Seoul, Republic of Korea; 11 Healthcare AI Research Institute, Seoul National University Hospital, Seoul, Korea

**Keywords:** CNS cancer, Cancer imaging

## Abstract

Adult diffuse gliomas are clinically and molecularly heterogeneous, complicating risk stratification and personalized management. We introduce GlioSurv, a multimodal transformer model based on an accelerated failure time framework to integrate multiparametric MRI, clinical and molecular variables, and treatment data for personalized survival prediction. In a retrospective analysis of 1944 patients, including one internal cohort (*n* = 891; mean OS 32.2 months) and three external cohorts (*n* = 84, 470, 499; mean OS 26.1, 18.8, 19.0 months), GlioSurv demonstrated robust discrimination (IAUC: 0.68–0.86), calibration (IBS: 0.10–0.21) and concordance (C-index: 0.61–0.80). It significantly outperformed a convolutional neural network, a vision transformer, and a non-imaging multimodal transformer (*p* < 0.01). Sequential integration of imaging, clinical, molecular, then treatment data, progressively improved C-index from 0.69 to 0.80 (*p* < 0.001). Interpretability analyses confirmed established prognostic factors and indicate the potential of GlioSurv to support personalized survival prediction and risk-stratified decision-making in diffuse glioma.

## Introduction

Adult-type diffuse gliomas, the most prevalent malignant primary brain tumors in adults, exhibit notable differences in prognosis both across and within molecularly defined subgroups^1,2^. The 2021 World Health Organization (WHO) classification categorizes these tumors into oligodendroglioma (isocitrate dehydrogenase [IDH]-mutant, 1p/19q-codeleted), astrocytoma (IDH-mutant, 1p/19q-intact), and glioblastoma (IDH-wildtype), each associated with distinct survival outcomes^3^. In particular, glioblastoma typically results in a median survival of only 15–18 months, even with intensive combination therapy^4,5^. In contrast, patients with IDH-mutant tumors often exhibit significantly longer survival^6^. These varying prognostic outcomes are further influenced by patient age, Karnofsky Performance Status (KPS), and extent of resection (EOR)^7^, emphasizing the clinical importance of precise, individualized survival estimation.

Accurate and personalized survival prediction in gliomas is crucial for optimizing treatment choices, informing patient counseling, stratifying clinical trial enrollment, and guiding follow-up imaging schedules^8–11^. Given the substantial prognostic heterogeneity driven by tumor biology, patient-specific factors, molecular alterations, and treatment responses^12–14^, accurate survival estimation requires integrative modeling of multimodal imaging, clinical, molecular, and treatment information^15–17^. Magnetic resonance imaging (MRI) is particularly valuable, offering noninvasive, high-resolution visualization of tumor morphology and signal variations, like the T2-FLAIR mismatch sign, which correlate with survival^18–20^. This highlights how combining radiological features with clinical and molecular information can significantly enhance risk stratification and support clinical decision-making.

Despite recent advances in glioma survival prediction using deep learning with integrated multimodal data, critical limitations remain. Prognostic accuracy is frequently compromised by missing or incomplete data, which are often absent due to high costs, clinical constraints, and limited resources. This not only restricts the applicability of deep learning approaches but also introduces concerns regarding selection bias^21,22^. The absence of treatment-related information is particularly problematic, as therapeutic strategies substantially influence survival probability^14,23,24^. Methodologically, many existing models rely on naive feature concatenation to integrate heterogeneous modalities, an approach that fails to capture complex, nonlinear interactions across data types^25^. Furthermore, grouping survival outcomes into broad risk categories limits the clinical usefulness of prognostic models, offering only categorical outputs instead of continuous probability estimates required for personalized decision-making^26^. These limitations highlight the need for more integrative and clinically applicable prognostic models.

Multimodal transformers^21,22,27^ have emerged as powerful architectures for integrating imaging, clinical, and molecular information. Transformers process multimodal data by modality-agnostic tokenization, leverage self-attention mechanisms for capturing intra-modal dependencies, and utilize cross-attention to effectively support inter-modal interactions, improving representation learning, understanding, classification, and prediction across multimodal tasks^28^. Recent studies in neuro-oncology have demonstrated that multimodal transformers effectively integrate MRI and clinical information, enhancing glioma molecular subtyping accuracy through improved feature fusion and interpretability^29^. Nevertheless, their direct application to predicting prognosis in glioma patients remains challenging due to the unique characteristics of glioma survival data, which involve accurately modeling both the occurrence and timing of patient mortality, as well as appropriately handling censoring cases where survival outcomes are not fully observed during the study period^30,31^. Thus, it is crucial to develop multimodal transformers specifically adapted to survival analysis to improve the accuracy, interpretability, and personalization of prognostic predictions in patients with adult-type diffuse gliomas.

Here, we present GlioSurv, a transformer-based framework for personalized survival prediction in adult-type diffuse glioma. The model encodes imaging, clinical, molecular, and treatment data through modality-specific transformers, followed by a decoder where survival tokens attend across modalities. To support time-to-event modelling, the decoder aligns with the Accelerated Failure Time (AFT) formulation^32^, enabling estimation of survival curves with uncertainty and attribution of prognostic contributions. In this multi-center study, predictive accuracy improved stepwise as data modalities were added, with treatment data providing additional gain. GlioSurv offers interpretable, patient-level survival estimates to support risk-stratified treatment planning in neuro-oncology.

## Results

### Cohort characteristics

We conducted a retrospective, multicenter analysis of 1944 adult-type diffuse glioma patients from four independent cohorts. These cohorts comprised an internal institutional cohort (*n* = 891) and three external validation cohorts: external institutional (*n* = 84), University of California San Francisco (UCSF; *n* = 470)^33^, and University of Pennsylvania (UPenn; *n* = 499)^34^. Table 1 provides details of baseline clinicopathological characteristics. The internal cohort was significantly younger (mean ± SD: 55.1 ± 14.6 years) than the UPenn cohort (63.0 ± 11.5 years). Patients at the external institutional (58.4 ± 14.2 years) and UCSF (56.8 ± 15.2 years) sites had intermediate ages (*P* < 0.001). Sex distribution was similar across all cohorts (*P* = 0.502). Mean overall survival (OS) also differed significantly (*P* < 0.001). OS was longest in the internal cohort (32.2 ± 35.4 months) and shortest at UCSF (18.8 ± 17.0 months), while the external institutional (26.1 ± 17.4 months) and UPenn (19.0 ± 21.5 months) cohorts exhibited intermediate OS. The baseline characteristics of the internal subsets, detailed in Supplementary Table 1, showed no significant imbalances that would compromise the internal validation.

**Table 1:** Baseline clinicopathological characteristics of adult-type diffuse glioma patients across four cohorts

Characteristics	Internal Institutional set (*n* = 891)	External validation sets			P value
		External institutional set (*n* = 84)	UCSF (*n* = 470)	UPenn (*n* = 499)	
Age	55.1 ± 14.6	58.4 ± 14.2	56.8 ± 15.2	63.0 ± 11.5	<0.001
Sex					0.502
Male	505/891 (56.7)	52/84 (61.9)	281/470 (59.8)	299/499 (59.9)	
Female	386/891 (43.3)	32/84 (38.1)	189/470 (40.2)	200/499 (40.1)	
Karnofsky performance status					<0.001
100	286/891 (32.1)	3/84 (3.6)	0/470 (0.0)	5/499 (1.0)	
90	257/891 (28.8)	16/84 (19.0)	0/470 (0.0)	37/499 (7.4)	
80	121/891 (13.6)	20/84 (23.8)	0/470 (0.0)	18/499 (3.6)	
70	89/891 (10.0)	22/84 (26.2)	0/470 (0.0)	6/499 (1.2)	
60	43/891 (4.8)	14/84 (16.7)	0/470 (0.0)	6/499 (1.2)	
50	22/891 (2.5)	7/84 (8.3)	0/470 (0.0)	0/499 (0.0)	
40	4/891 (0.4)	1/84 (1.2)	0/470 (0.0)	2/499 (0.4)	
**30**	1/891 (0.1)	1/84 (1.2)	0/470 (0.0)	1/499 (0.2)	
Unknown	68/891 (7.6)	0/84 (0.0)	470/470 (100.0)	424/499 (85.0)	
WHO Grade					<0.001
Grade 2	73/891 (8.2)	7/84 (8.3)	46/470 (9.8)	0/499 (0.0)	
Grade 3	137/891 (15.4)	11/84 (13.1)	29/470 (6.2)	0/499 (0.0)	
Grade 4	681/891 (76.4)	66/84 (78.6)	395/470 (84.0)	499/499 (100.0)	
Histopathology					<0.001
Glioblastoma, IDH wildtype	638/891 (71.6)	66/84 (78.6)	367/470 (78.1)	499/499 (100.0)	
Astrocytoma, IDH-mutant	118/891 (13.2)	12/84 (14.3)	90/470 (19.1)	0/499 (0.0)	
Oligodendroglioma, IDH-mutant, and 1p/19q-codeleted	135/891 (15.2)	6/84 (7.1)	13/470 (2.8)	0/499 (0.0)	
IDH mutation					<0.001
Wildtype	638/891 (71.6)	68/84 (81.0)	367/470 (78.1)	499/499 (100.0)	
Mutated	253/891 (28.4)	16/84 (19.0)	103/470 (21.9)	0/499 (0.0)	
1p/19q co-deletion					<0.001
Non-codeleted	717/891 (80.5)	76/84 (90.5)	364/470 (77.4)	0/499 (0.0)	
Codeleted	144/891 (16.2)	8/84 (9.5)	15/470 (3.2)	0/499 (0.0)	
Unknown	30/891 (3.4)	0/84 (0.0)	91/470 (19.4)	499/499 (100.0)	
MGMTp methylation					<0.001
Unmethylated	382/891 (42.9)	51/84 (60.7)	108/470 (23.0)	151/499 (30.3)	
Methylated	493/891 (55.3)	33/84 (39.3)	283/470 (60.2)	104/499 (20.8)	
Unknown	16/891 (1.8)	0/84 (0.0)	79/470 (16.8)	244/499 (48.9)	
Extent of resection					<0.001
Gross total resection	551/891 (61.8)	54/84 (64.3)	242/470 (51.5)	292/499 (58.5)	
Subtotal resection	221/891 (24.8)	20/84 (23.8)	183/470 (38.9)	183/499 (36.7)	
Biopsy	112/891 (12.6)	10/84 (11.9)	45/470 (9.6)	0/499 (0.0)	
Unknown	7/891 (0.8)	0/84 (0.0)	0/470 (0.0)	24/499 (4.8)	
Radiation therapy					<0.001
Received	662/891 (74.3)	0/84 (0.0)	0/470 (0.0)	0/499 (0.0)	
Not Received	229/891 (25.7)	84/84 (100.0)	470/470 (100.0)	499/499 (100.0)	
Unknown					
Chemotherapy					<0.001
Received	555/891 (62.3)	0/84 (0.0)	0/470 (0.0)	0/499 (0.0)	
Not Received	336/891 (37.7)	84/84 (100.0)	470/470 (100.0)	499/499 (100.0)	
Unknown					
Overall survival (Months)	32.2 ± 35.4	26.1 ± 17.4	18.8 ± 17.0	19.0 ± 21.5	<0.001
Death					<0.001
Occurred	509/891 (57.1)	60/84 (71.4)	234/470 (49.8)	482/499 (96.6)	
Censored	382/891 (42.9)	24/84 (28.6)	236/470 (50.2)	17/499 (3.4)	

Continuous variables are presented as mean ± standard deviation, and categorical variables as n (%). P-values were calculated using one-way analysis of variance for continuous variables and chi-square test for categorical variables. Overall survival was compared using a multivariate log-rank test.

*GTR* gross total resection, *IDH* isocitrate dehydrogenase, *KPS* Karnofsky Performance Status, *MGMTp* O6-methylguanine-DNA methyltransferase promoter, *UCSF* University of California, San Francisco, *UPenn* University of Pennsylvania, *WHO* World Health Organization.

Significant differences among cohorts were evident for KPS, WHO tumor grade, histopathological subtype, IDH mutation status, 1p/19q codeletion, O6-methylguanine-DNA methyltransferase promoter (MGMTp) methylation, EOR, radiotherapy treatment, and chemotherapy treatment (all *P* < 0.001). Kaplan–Meier survival analyses, when stratified by these variables, revealed pronounced OS differences (log-rank *P* < 0.001 for all factors except sex [*P* = 0.03]). These findings underscore the importance of integrating diverse prognostic factors within a unified model (Supplementary Fig. 1).

### Model performance

**Fig. 1:**
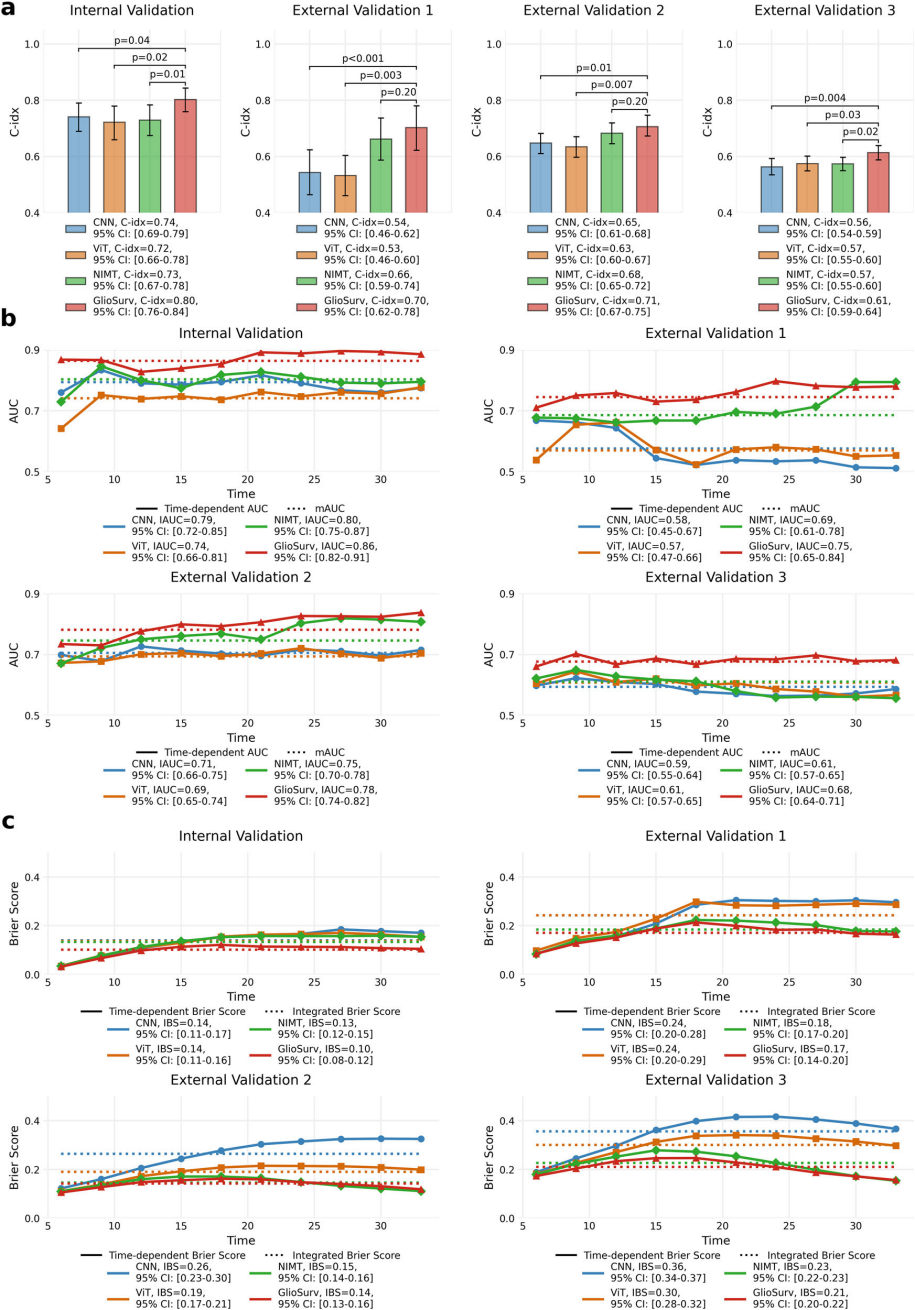
Comparative performance of GlioSurv and benchmark models across validation cohorts. **a** Mean concordance index (C-index) with 95% confidence intervals (CIs) for convolutional neural network (CNN), vision transformer (ViT), Non-Imaging Multimodal Transformer (NIMT), and GlioSurv across the internal institutional, external institutional, University of California San Francisco (UCSF), and University of Pennsylvania (UPenn) cohorts. P-values, derived from two-sample bootstrap tests, compare each benchmark model against GlioSurv. **b** Time-dependent area under the receiver-operating characteristic curve (AUC) over 36 months. Solid lines represent AUC at each timepoint with shaded 95% CIs; dashed horizontal lines indicate the integrated AUC (IAUC). **c** Time-dependent Brier score over 36 months; lower values signify better calibration.

We evaluated prognostic performance using three complementary survival metrics: discrimination, assessed by the integrated area under the time-dependent receiver-operating characteristic curve (IAUC); calibration, measured by the integrated Brier score (IBS); and ranking concordance, determined by the concordance index (C-index). We systematically compared four model architectures across all cohorts: convolutional neural network (CNN), vision transformer (ViT), Non-Imaging Multimodal Transformer (NIMT), and GlioSurv (Table 2 and Fig. 1).

**Table 2:** Performance comparison of survival prediction models across validation cohorts

Center	Metric	(1) CNN	(2) ViT	(3) NIMT	(4) GlioSurv	P value (1) vs (4)	P value (2) vs (4)	P value (3) vs (4)
Internal validation	IAUC	0.79 [0.72, 0.85]	0.74 [0.66, 0.81]	0.80 [0.75, 0.87]	**0.86 [0.82, 0.91]**	<0.001	<0.001	<0.001
	IBS	0.14 [0.11, 0.17]	0.13 [0.11, 0.16]	0.13 [0.12, 0.15]	**0.10 [0.08, 0.12]**	<0.001	<0.001	<0.001
	C-index	0.74 [0.69, 0.79]	0.72 [0.66, 0.78]	0.73 [0.67, 0.78]	**0.80 [0.76, 0.84]**	0.045	0.019	0.015
External validation 1	IAUC	0.58 [0.45, 0.67]	0.57 [0.47, 0.66]	0.69 [0.61, 0.78]	**0.75 [0.65, 0.84]**	<0.001	<0.001	<0.001
	IBS	0.24 [0.20, 0.28]	0.24 [0.20, 0.29]	0.18 [0.17, 0.20]	**0.17 [0.14, 0.20]**	<0.001	<0.001	<0.001
	C-index	0.54 [0.46, 0.62]	0.53 [0.46, 0.60]	0.66 [0.59, 0.74]	**0.70 [0.62, 0.78]**	<0.001	0.003	0.204
External validation 2	IAUC	0.71 [0.66, 0.75]	0.69 [0.65, 0.74]	0.75 [0.70, 0.78]	**0.78 [0.74, 0.82]**	<0.001	<0.001	<0.001
	IBS	0.26 [0.23, 0.30]	0.19 [0.17, 0.21]	**0.14 [0.14, 0.16]**	**0.14 [0.13, 0.16]**	<0.001	<0.001	<0.001
	C-index	0.65 [0.61, 0.68]	0.63 [0.60, 0.67]	0.68 [0.65, 0.72]	**0.71 [0.67, 0.75]**	0.015	0.007	0.205
External validation 3	IAUC	0.59 [0.55, 0.64]	0.61 [0.57, 0.65]	0.61 [0.57, 0.65]	**0.68 [0.64, 0.71]**	<0.001	<0.001	<0.001
	IBS	0.35 [0.34, 0.37]	0.29 [0.28, 0.32]	0.22 [0.22, 0.23]	**0.21 [0.20, 0.22]**	<0.001	<0.001	<0.001
	C-index	0.56 [0.54, 0.59]	0.57 [0.55, 0.60]	0.57 [0.55, 0.60]	**0.61 [0.59, 0.64]**	0.004	0.028	0.019

Values are presented as metric [95% confidence interval]. Bold values indicate the best performing model for each metric. P-values were calculated using two-sample bootstrap tests comparing each benchmark model against GlioSurv.

*C-index* concordance index, *CNN* convolutional neural network, *ViT* vision transformer, *NIMT* Non-Imaging Multimodal Transformer, *IAUC* integrated area under the time-dependent receiver operating characteristic curve, *IBS* integrated Brier score, *UCSF* University of California, San Francisco, *UPenn* University of Pennsylvania.

In the internal validation cohort, GlioSurv achieved an IAUC of 0.86 [0.82, 0.91], an IBS of 0.10 [0.08, 0.12], and a C-index of 0.80 [0.76, 0.84]. GlioSurv demonstrated substantial improvements compared with baseline architectures. Specifically, for CNN (IAUC, 0.79 [0.72, 0.85]; IBS, 0.14 [0.11, 0.17]; C-index, 0.74 [0.69, 0.79]), ViT (IAUC, 0.74 [0.66, 0.81]; IBS, 0.13 [0.11, 0.16]; C-index, 0.72 [0.66, 0.78]), and NIMT (IAUC, 0.80 [0.75, 0.87]; IBS, 0.13 [0.12, 0.15]; C-index, 0.73 [0.67, 0.78]), the differences in IAUC and IBS were all significant (*P* < 0.001), and C-index differences were also significant (CNN: *P* = 0.045; ViT: *P* = 0.019; NIMT: *P* = 0.015) (Fig. 1a, left).

In the external institutional cohort, GlioSurv showed an IAUC of 0.75 [0.65, 0.84], an IBS of 0.17 [0.14, 0.20], and a C-index of 0.70 [0.62, 0.78]. Compared to CNN (IAUC, 0.58 [0.45, 0.67]; IBS, 0.24 [0.20, 0.28]; C-index, 0.54 [0.46, 0.62]) and ViT (IAUC, 0.57 [0.47, 0.66]; IBS, 0.24 [0.20, 0.29]; C-index, 0.53 [0.46, 0.60]), GlioSurv showed significantly better IAUC and IBS (*P* < 0.001 for both metrics), and higher C-index (CNN: *P* < 0.001; ViT: *P* = 0.003). Compared to NIMT (IAUC, 0.69 [0.61, 0.78]; IBS, 0.18 [0.17, 0.20]; C-index, 0.66 [0.59, 0.74]), GlioSurv showed significant improvements in IAUC and IBS (*P* < 0.001), but the C-index difference was not statistically significant (*P* = 0.204) (Fig. 1a, center-left).

At UCSF, GlioSurv recorded an IAUC of 0.78 [0.74, 0.82], an IBS of 0.14 [0.13, 0.16], and a C-index of 0.71 [0.67, 0.75]. When compared to CNN (IAUC, 0.71 [0.66, 0.75]; IBS, 0.26 [0.23, 0.30]; C-index, 0.65 [0.61, 0.68]) and ViT (IAUC, 0.69 [0.65, 0.74]; IBS, 0.19 [0.17, 0.21]; C-index, 0.63 [0.60, 0.67]), GlioSurv showed significantly better IAUC and IBS (*P* < 0.001 for both metrics), and higher C-index (CNN: *P* = 0.015; ViT: *P* = 0.007). Compared to NIMT (IAUC, 0.75 [0.70, 0.78]; IBS, 0.14 [0.14, 0.16]; C-index, 0.68 [0.65, 0.72]), GlioSurv again showed significant improvement in IAUC and IBS (*P* < 0.001), but not in C-index (*P* = 0.205) (Fig. 1a, center-right).

In the UPenn cohort, which included only glioblastoma patients, GlioSurv achieved an IAUC of 0.68 [0.64, 0.71], an IBS of 0.21 [0.20, 0.22], and a C-index of 0.61 [0.59, 0.64]. Compared to CNN (IAUC, 0.59 [0.55, 0.64]; IBS, 0.35 [0.34, 0.37]; C-index, 0.56 [0.54, 0.59]), ViT (IAUC, 0.61 [0.57, 0.65]; IBS, 0.29 [0.28, 0.32]; C-index, 0.57 [0.55, 0.60]), and NIMT (IAUC, 0.61 [0.57, 0.65]; IBS, 0.22 [0.22, 0.23]; C-index, 0.57 [0.55, 0.60]), GlioSurv demonstrated significantly better IAUC and IBS (*P* < 0.001 for all), and higher C-index (CNN: *P* = 0.004; ViT: *P* = 0.028; NIMT: *P* = 0.019) (Fig. 1a, far right).

Time-dependent analyses revealed temporal performance patterns across architectures (Fig. 1b, c). Both GlioSurv and NIMT maintained stable AUC values from 6 to 36 months of follow-up, whereas CNN and ViT exhibited a decline in discrimination over time. For calibration, the Brier score curves of ViT showed a gradual increase, indicating worsening calibration, while CNN demonstrated an even steeper rise. In contrast, GlioSurv and NIMT consistently maintained low and stable Brier scores. In addition, GlioSurv and NIMT preserved stable AUC and Brier trajectories across all external validation cohorts, while the other architectures showed progressive performance decline and increasing calibration errors. To further assess calibration at clinically relevant timepoints, we analyzed the calibration slope and intercept. The GlioSurv model demonstrated good calibration at both 6 and 12 months across all cohorts, with trendlines closely approximating the ideal diagonal line (Supplementary Fig. 2). Specifically, at 12 months, the calibration slope was 1.13 in the internal cohort and ranged from 0.83 to 0.98 in the external cohorts, indicating a strong agreement between predicted and observed survival probabilities.

### Risk stratification by Kaplan–Meier analysis

**Fig. 2:**
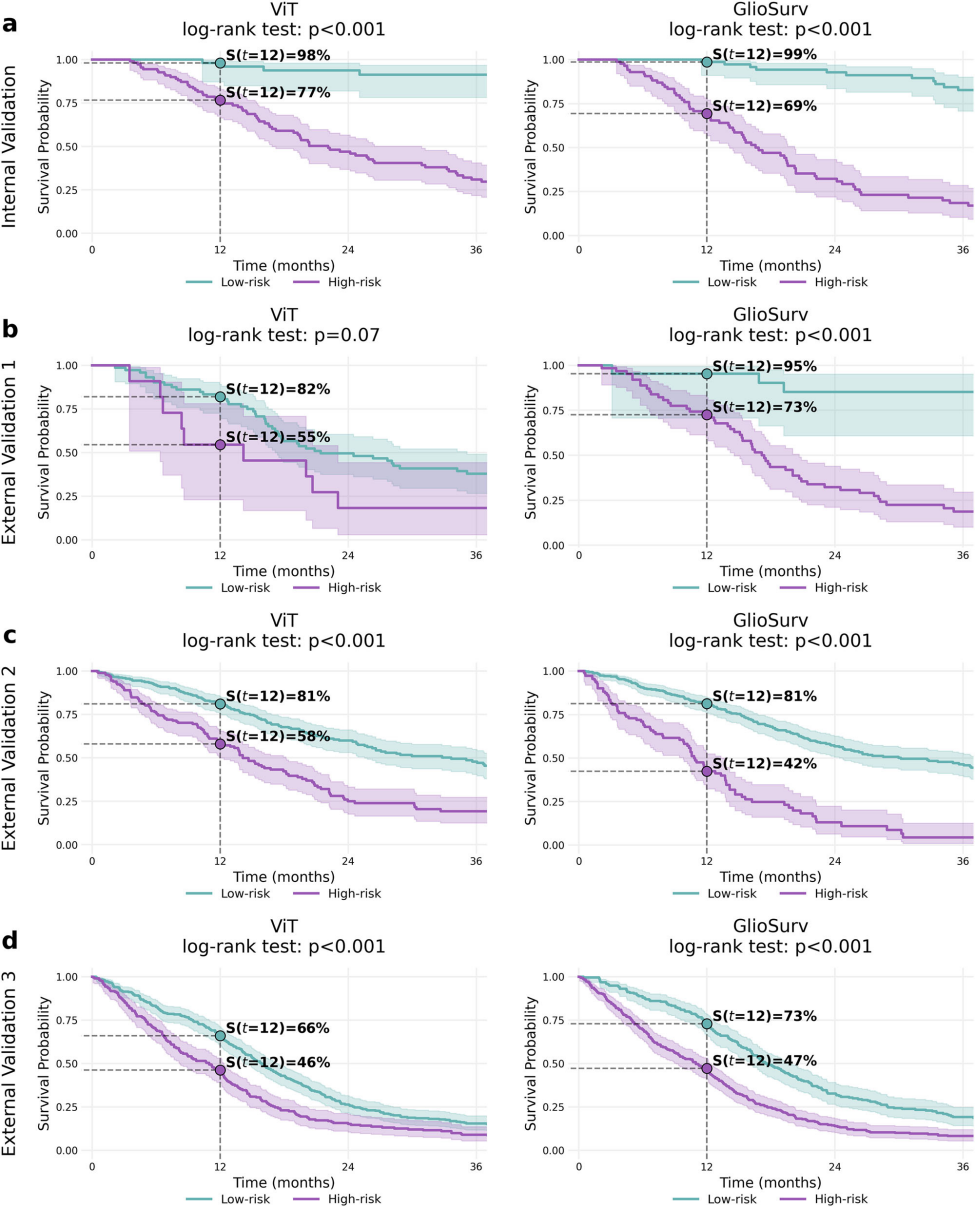
Risk stratification by ViT (image-only) versus GlioSurv (imaging + clinical) using different data modalities. Kaplan–Meier survival curves for patients stratified into low-risk (blue lines) and high-risk (red lines) groups based on the median predicted risk score from each model. Panels display results for: **a** internal institutional cohort, **b** external institutional cohort, **c** University of California San Francisco (UCSF) cohort, and **d** University of Pennsylvania (UPenn) cohort. Within each panel, comparisons are shown for Vision Transformer (ViT) (left) and GlioSurv (right). Vertical dashed lines mark the 12-month timepoint, with corresponding survival probabilities annotated. Log-rank P-values assess the significance of separation between risk groups for each model.

To assess the clinical utility of model-derived risk scores, we stratified patients into high- and low-risk groups using an optimal cutoff determined by the log-rank test. Kaplan–Meier survival curves were then constructed for each validation cohort (Fig. 2 and Supplementary Fig. 3).

In the internal validation cohort, all four architectures significantly differentiated risk strata (log-rank *P* < 0.001). GlioSurv showed clear separation between high- and low-risk groups, with estimated 12-month survival probabilities of 99% for the low-risk group and 69% for the high-risk group. For ViT, the 12-month survival probabilities were 98% (low-risk) and 77% (high-risk); for CNN, 97% and 72%; and for NIMT, 89% and 47%, respectively.

In the external institutional cohort, GlioSurv demonstrated significant separation (*P* < 0.001), with 12-month survival probabilities of 95% (low-risk) and 73% (high-risk). ViT did not achieve statistical significance (*P* = 0.07), while CNN showed 81% (low-risk) and 62% (high-risk) (*P* = 0.03). NIMT also demonstrated significant stratification, with 100% (low-risk) and 73% (high-risk) (*P* < 0.001).

In the UCSF cohort, all models showed statistically significant separation (all *P* < 0.001). For GlioSurv, the 12-month survival probabilities were 81% (low-risk) and 42% (high-risk); for ViT, 81% and 58%; for CNN, 79% and 44%; and for NIMT, 99% and 65%.

In the UPenn cohort, GlioSurv exhibited significant separation (*P* < 0.001), with 12-month survival probabilities of 73% (low-risk) and 47% (high-risk). ViT showed 66% and 46%, CNN 62% and 42%, and NIMT 67% and 43% for low- and high-risk groups, respectively; all these models also achieved statistical significance (*P* < 0.001).

### Subgroup performance across cohorts

We conducted several subgroup analyses to evaluate the robustness of GlioSurv’s performance across different patient cohorts. A key finding was that the model’s predictive accuracy was significantly higher in subgroups with complete clinical and molecular data compared to those with incomplete data (Supplementary Table 2, all *p* < 0.001).

The model’s performance also varied across clinically relevant subgroups (Supplementary Tables 3–9). Generally, GlioSurv demonstrated stronger predictive accuracy in younger patients (age 15-47), individuals with a higher functional status (KPS 80-100), and in tumors with MGMTp methylation. For instance, the model’s C-index was significantly higher for patients with MGMTp-methylated tumors compared to unmethylated ones in both the internal validation (0.83 vs. 0.68, *p* = 0.012) and the first external validation cohort (0.80 vs. 0.59, *p* < 0.001). Conversely, performance tended to be lower in older patients and those with poorer functional status. Due to an insufficient number of events in certain subgroups, such as lower-grade tumors or specific histopathologies, robust performance estimation was not always possible, as indicated by ‘N/A’ values in the supplementary tables.

### Incremental integration of prognostic information

To quantify the incremental benefit of each prognostic factor, we conducted an incremental analysis in the internal institutional cohort. This analysis progressively incorporated clinical and molecular variables into a baseline fusion of multiparametric MRI and patient demographics (M + P). The initial M + P model yielded IAUC of 0.71 [0.64, 0.80], IBS of 0.13 [0.11, 0.15], and C-index of 0.69 [0.64, 0.75]. With the addition of KPS (M + *P* + K), discrimination remained unchanged (IAUC, 0.71 [0.66, 0.81]), but calibration (IBS, 0.12 [0.11, 0.15]) and concordance (C-index, 0.71 [0.65, 0.76]) improved. Incorporating genetic markers—IDH mutation, 1p/19q co-deletion, and MGMTp methylation (M + *P* + K + G)—resulted in IAUC of 0.81 [0.76, 0.87], IBS of 0.11 [0.09, 0.13], and C-index of 0.77 [0.72, 0.81]. The full model (M + *P* + K + G + T), which additionally included treatment variables, reached IAUC of 0.86 [0.82, 0.91], IBS of 0.10 [0.08, 0.12], and C-index of 0.80 [0.76, 0.84]. Compared to the baseline M + P model, the comprehensive model demonstrated significant improvements across all metrics (all *P* < 0.001); however, the C-index difference between the M + *P* + K + G model and the full model was not statistically significant (*P* = 0.127) (Table 3).

**Table 3:** Incremental performance improvement with sequential addition of prognostic information for GlioSurv in the internal institutional cohort

Metric	(1) GlioSurv (M + P)	(2) GlioSurv (M + *P* + K)	(3) GlioSurv (M + *P* + K + G)	(4) GlioSurv (M + *P* + K + G + T)	P value (1) vs (4)	P value (2) vs (4)	P value (3) vs (4)
IAUC	0.71 [0.64, 0.80]	0.71 [0.66, 0.81]	0.81 [0.76, 0.87]	**0.86 [0.82, 0.91]**	<0.001	<0.001	<0.001
IBS	0.13 [0.11, 0.15]	0.12 [0.11, 0.15]	0.11 [0.09, 0.13]	**0.10 [0.08, 0.12]**	<0.001	<0.001	<0.001
C-index	0.69 [0.64, 0.75]	0.71 [0.65, 0.76]	0.77 [0.72, 0.81]	**0.80 [0.76, 0.84]**	<0.001	0.004	0.127

Values are presented as metric [95% confidence interval]. Bold values indicate the full GlioSurv (M + *P* + K + G + T) model, representing the configuration with all prognostic information. P-values were calculated using two-sample bootstrap tests comparing each reduced model variant against the full GlioSurv (M + *P* + K + G + T) model.

*C-index* concordance index, *G* genetic markers (IDH mutation status, 1p/19q codeletion status, MGMTp methylation status), *IAUC* integrated area under the time-dependent receiver operating characteristic curve, *IBS* integrated Brier score, *IDH* isocitrate dehydrogenase, *K* Karnofsky Performance Status, *M* multiparametric magnetic resonance imaging, *MGMTp* O6-methylguanine-DNA methyltransferase promoter, *P* patient demographics (age, sex), *T* treatment data (extent of resection, radiotherapy, chemotherapy).

**Fig. 3:**
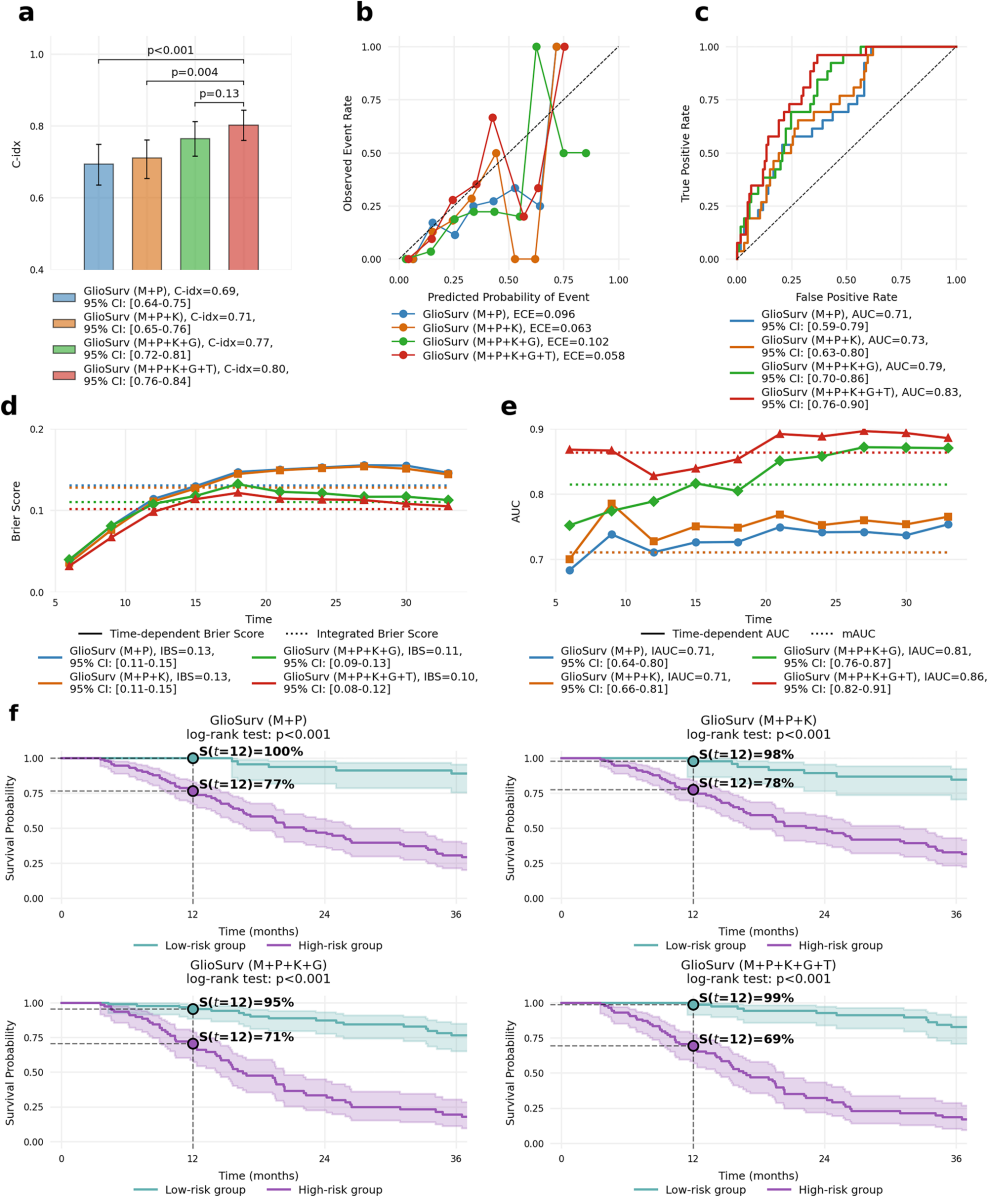
Impact of incremental multimodal data integration on GlioSurv performance in the internal institutional cohort. Performance metrics for GlioSurv variants with sequential addition of prognostic information: M, multiparametric magnetic resonance imaging; P, patient demographics (age, sex); K, Karnofsky Performance Status; G, genetic markers (isocitrate dehydrogenase [IDH] mutation status, 1p/19q codeletion status, O6-methylguanine-DNA methyltransferase promoter [MGMTp] methylation status); T, treatment information (extent of resection, radiotherapy, chemotherapy). **a** Concordance index (C-index). **b** Calibration curves plotting observed event rates against predicted probabilities; the diagonal line represents perfect calibration. Inset values indicate Expected Calibration Error (ECE). **c** Receiver-operating characteristic (ROC) curves with area under the curve (AUC) values. **d** Time-dependent Brier score over 36 months. **e** Time-dependent AUC over 36 months. Shaded areas in (**f**) Represent 95% confidence intervals.

Calibration plots (Fig. 3b) show a reduction in expected calibration error, from 0.096 in the M + P model to 0.058 in the full M + *P* + K + G + T model. The 12-month survival prediction AUC curves (Fig. 3c) illustrate improvements in discrimination with each additional prognostic factor. Time-dependent Brier score curves (Fig. 3d) demonstrate that the addition of clinical, genetic, and treatment variables progressively reduced prediction error over time, with the full model maintaining the lowest Brier scores throughout follow-up. Similarly, time-dependent AUC curves (Fig. 3e) indicate that discrimination improved at all time points as more prognostic factors were integrated, with the comprehensive model consistently achieving the highest AUC values. Kaplan–Meier stratification showed that at 12 months, the predicted low- versus high-risk survival changed from 77% versus 100% in the M + P model to 69% versus 99% in the full model (Fig. 3f).

### Patient-level interpretability analysis

**Fig. 4:**
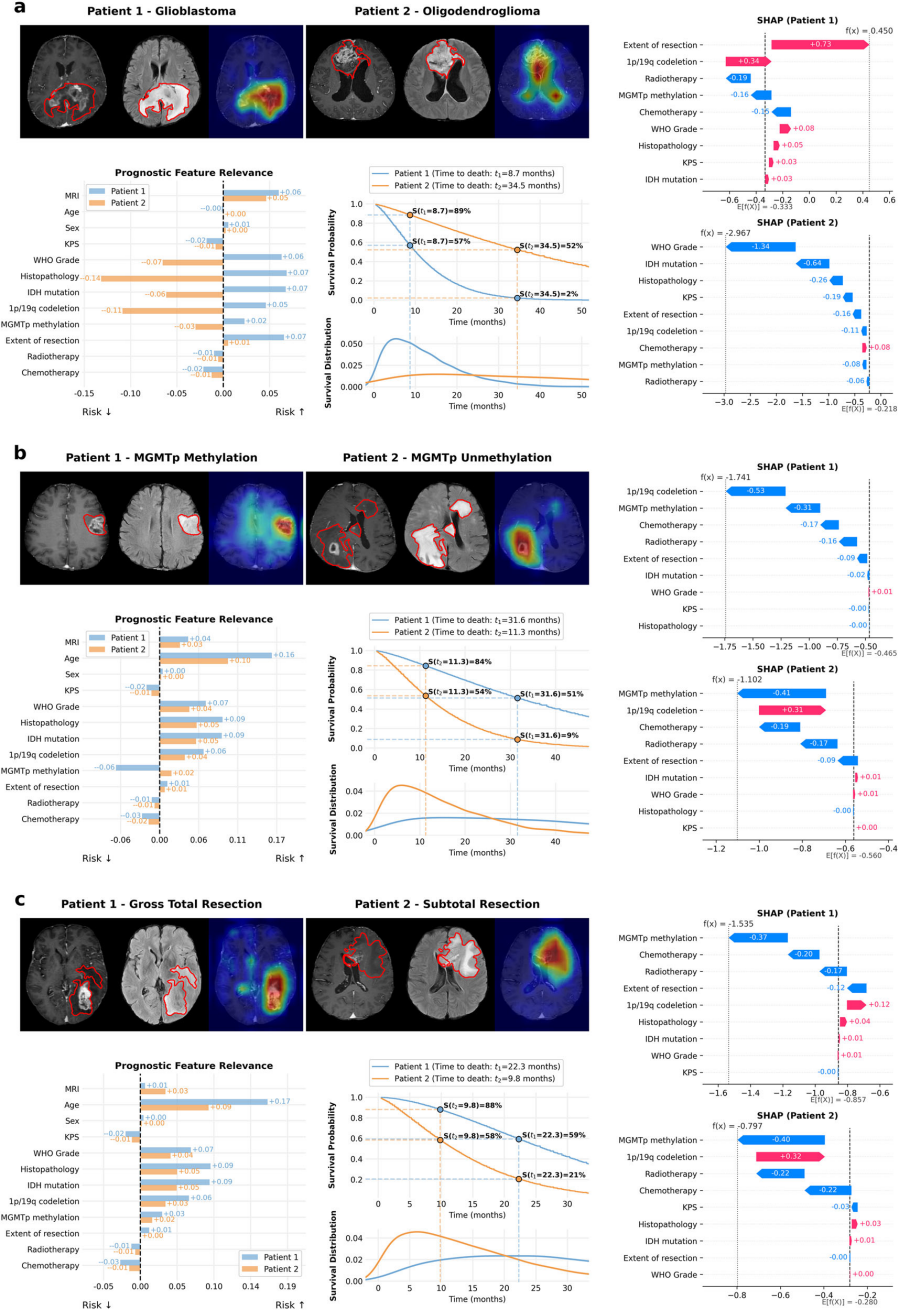
Patient-level interpretability of GlioSurv predictions. Case-based visualizations illustrating GlioSurv’s predictive mechanisms for three pairs of patients, each differing by a key prognostic factor. **a** Histopathological subtype: glioblastoma (left) versus oligodendroglioma (right). **b** O6-methylguanine-DNA methyltransferase promoter (MGMTp) methylation status: methylated (left) versus unmethylated (right). **c** Extent of resection: gross total resection (left) versus subtotal resection (right). For each patient: Left panels show axial magnetic resonance imaging (MRI) slices with tumor segmentation outlines (red) and overlaid Eigen-Class Activation Mapping (Eigen-CAM) activation heatmaps (Red colors indicate regions of higher influence on prediction). Middle panels display feature relevance scores for key clinical, molecular, and imaging variables; positive scores (red bars) indicate contribution to increased predicted risk, while negative scores (blue bars) indicate protective effects. Right panels present individualized survival probability curves (top sub-panel), with the 12-month survival probability annotated, and predicted survival time density curves (bottom sub-panel), illustrating the probability distribution of predicted survival times, and SHAP summary plots illustrating the contribution of each feature to the model output for each patient.

To qualitatively evaluate the model’s prognostic reasoning, we visualized attention-derived activation maps for representative patient pairs stratified by key clinical factors: histopathological subtype (glioblastoma vs. oligodendroglioma), MGMTp methylation (methylated vs. unmethylated), and extent of resection (gross total vs. subtotal) (Fig. 4). These activation maps were computed by aggregating self-attention weights from the transformer backbone, thereby localizing imaging regions most influential to the predicted risk. In the glioblastoma case, relevance activations were predominantly localized to the enhancing tumor core and peritumoral edema, whereas in the oligodendroglioma case, activations were more diffuse and peripherally distributed (Fig. 4a). In the MGMTp unmethylated patient, the model emphasized residual contrast-enhancing tissue (Fig. 4b), while in the subtotal resection case, attention extended beyond the resection cavity into surrounding FLAIR hyperintensities (Fig. 4c). These findings demonstrate that GlioSurv attends to spatially distinct, high-risk imaging features across clinically meaningful subgroups. However, these patterns were not uniformly observed across all individuals, with inter-individual variability noted in the localization of relevance activations by the attention-based deep learning model.

To quantitatively assess the spatial reliability of these activation maps, we evaluated their overlap with ground-truth tumor segmentations. The model demonstrated high localization accuracy across all validation cohorts, correctly identifying the tumor region in 73–94% of cases (Supplementary Table 10 and Supplementary Fig. 4). While pixel-level sensitivity was modest (0.31–0.45), reflecting the sparse nature of attention maps, the AUC for pixel-level classification was high (0.84–0.97). Crucially, this localization accuracy was strongly correlated with the model’s predictive reliability. Cases with accurate tumor localization demonstrated significantly higher pixel-level AUCs compared to cases with inaccurate localization, where performance dropped to near-random levels (e.g., AUC 0.97 vs. 0.51 in the internal cohort; *p* < 0.001 for all cohorts) (Supplementary Table 11 and Supplementary Fig. 5).

Feature relevance scores were also derived from transformer attention weights, aggregated across input modalities, and presented as horizontal bar plots to quantify the contribution of each prognostic factor—clinical, molecular, and imaging—to the model’s survival prediction. In the histopathology comparison, protective factors included oligodendroglioma histology (–0.14), IDH mutation (–0.06), and 1p/19q codeletion (–0.11). Conversely, glioblastoma subtype (+0.07) and IDH wildtype status (+0.07) were associated with elevated predicted risk. For MGMTp status, older age emerged as the strongest contributor (+0.16 in unmethylated vs. +0.10 in methylated), while methylation exerted a protective effect (–0.06 vs. +0.02). In the EOR comparison, age (+0.17 vs. +0.09) and IDH status (+0.09 vs. +0.05) contributed most substantially, whereas resection extent showed minimal direct attribution (+0.01 vs. 0.00), potentially due to its collinearity with residual imaging features (Supplementary Tables 12–14).

To visualize patient-specific prognostic trajectories, we generated individualized survival probability curves and risk distributions (Fig. 4, center bottom). These visualizations are accompanied by SHapley Additive exPlanations (SHAP)^35^ plots, which illustrate the specific contributions of each feature to the final prediction for each individual. These probabilistic density curves illustrate individualized survival risks over time: the glioblastoma patient had a predicted 57% survival probability at 8.7 months, while the oligodendroglioma patient exhibited an 89% probability at 34.5 months. The MGMTp unmethylated patient demonstrated a narrow distribution centered before 12 months, whereas the methylated counterpart showed a broader, right-skewed distribution, reflecting greater prognostic uncertainty.

## Discussion

In this study, we developed and validated GlioSurv, a novel multimodal transformer model. GlioSurv integrates multiparametric MRI with structured clinical and molecular features and treatment information, employing an AFT framework^32^. This approach uses masked cross-attention to handle incomplete data and directly estimates patient-specific survival metrics. Evaluated on 1944 adult-type diffuse glioma patients from four independent cohorts, GlioSurv achieved superior performance compared to CNN, ViT, and NIMT models. This enhanced performance was demonstrated by significant improvements in the IAUC (up to 0.18), reductions in the IBS (up to 0.14), and increases in the C-index (up to 0.17) across model comparisons (all *P* < 0.001 for IAUC and IBS), though C-index improvements over NIMT were not statistically significant in two cohorts (*P* = 0.204, 0.205). A critical distinction was that while NIMT, which uses only clinical, genetic, and treatment data without imaging, maintained relatively stable performance across external datasets due to its discrete input features, this same characteristic limited its ability to provide precise, personalized risk stratification. Time-dependent analyses confirmed GlioSurv’s stable discrimination and calibration over 36 months. Furthermore, Kaplan–Meier stratification revealed significant survival differences between model-defined high- and low-risk groups (*P* < 0.001). These findings collectively underscore GlioSurv’s robust prognostic capabilities and its generalizability across diverse imaging protocols, demographic profiles, and clinical settings.

Importantly, these results highlight the added prognostic value of integrating multiple data modalities into a unified survival model. Classical prognostic factors—such as patient age, KPS, EOR, IDH mutation status, and MGMTp methylation—are well established as independent survival predictors in adult-type diffuse gliomas^5,6,12,36,37^. Consistent with this literature, we observed stepwise performance enhancements when sequentially adding clinical, molecular, and finally treatment variables, with both C-index (from 0.69 to 0.80, *p* < 0.001) and IAUC (from 0.71 to 0.86, *p* < 0.001) showing significant improvements. This pattern reflects the complex nonlinear interplay among these modalities, interactions that single-modality models typically fail to capture. In particular, including treatment variables yielded substantial improvements in both IAUC (a 0.05 increase) and C-index (a 0.03 increase), highlighting treatment as a critical prognostic factor with potential to improve survival modeling. This suggests that models without treatment information may potentially underestimate or overestimate survival for patients under various treatment approaches.

Current glioma survival prediction studies present several limitations that restrict their clinical application and demonstrate the need for improved methodologies. Prior CNN-based models primarily incorporated imaging information^38,39^. Recent multimodal survival prediction studies also excluded detailed treatment data essential for accurate prognostication, including EOR, radiotherapy, and chemotherapy information^40–42^. This exclusion reflects a broader methodological challenge associated with missing data, as many studies require complete inputs, which are rarely available in clinical settings and are often insufficiently addressed. Without adequate resolution, this challenge can lead to biased estimates and compromise the broader applicability of the prediction model^21,22^. While some transformer-based models have begun utilizing attention mechanisms, many continue to use basic feature concatenation, limiting their ability to capture complex nonlinear interactions^42–44^. Additionally, conventional survival models, including Cox regression and binary survival classification models, offer limited quantification of predictive uncertainty. These approaches primarily capture group-level patterns rather than individual-level prognoses, making it challenging to generate personalized survival estimates with appropriate confidence intervals. These limitations underscore the need for an approach that incorporates comprehensive treatment modeling, effective missing-data handling, advanced cross-modal integration, and patient-specific predictions.

GlioSurv offers solutions to these limitations through four key approaches. First, the explicit incorporation of treatment variables such as extent of resection, radiotherapy, and chemotherapy resulted in a measurable improvement in predictive accuracy. Second, by modeling nonlinear interactions across imaging, clinical, and molecular data, our masked cross-attention approach adaptively handles missing features and addresses the limitations of static feature fusion strategies. Third, adopting an AFT framework enables direct estimation of individualized survival distributions with uncertainty quantification, offering richer prognostic information than conventional models limited to static risk groups or cohort-level metrics. Finally, the attention weights in GlioSurv enable interpretable visualization of modality-level contributions to individual predictions.

The interpretability features of GlioSurv address an important challenge to the clinical adoption of AI-based prognostic models. Using feature attribution techniques such as Eigen-Class Activation Mapping (Eigen-CAM)^45^ Gradient-weighted Class Activation Mapping (Grad-CAM)^46^ feature attribution analysis, and SHapley Additive exPlanations (SHAP)^35^, GlioSurv offers transparent insights into its decision-making process. These visualizations reflect established prognostic hierarchies in gliomas, including shorter predicted survival for glioblastoma and IDH wild-type cases, and longer survival for patients with IDH mutations and 1p/19q codeletion, characteristic of oligodendroglioma^3,6^. Consistent with prior clinical evidence, MGMTp methylation emerged as a dominant protective factor, and increasing age further influenced risk stratification^14,36^. Furthermore, GlioSurv’s spatial attention maps delineate intratumoral regions of prognostic relevance, potentially reflecting underlying microenvironmental variations in cellularity and vascularity^47,48^, but histopathologic correlation is needed to validate these spatial patterns. These multimodal and spatially-explicit visualizations enhance model interpretability and suggest correspondence with radiologic features relevant to tumor stratification, supporting their potential integration into prospective validation workflows.

The clinical implementation and future development of GlioSurv present both opportunities and challenges. GlioSurv generates individualized survival curves with confidence intervals, enabling communication of patient-specific prognostic probabilities at clinically meaningful timepoints, rather than relying on population-level averages^49^. To formally assess its potential impact on clinical decision-making, we conducted a decision curve analysis. This analysis demonstrated that for predicting 12-month mortality, GlioSurv offered a superior net benefit compared to the default strategies of treating all or no patients across a wide range of clinically relevant risk thresholds in all validation cohorts (Supplementary Fig. 6). By providing patient-specific predictions, GlioSurv has the potential to facilitate customized treatment planning, enabling clinicians to adjust treatment intensity based on individual risk profiles^5^. Patients predicted to have longer survival may benefit from more intensive interventions or enrollment in clinical trials. In contrast, those with shorter predicted survival may require early discussions about supportive care and quality-of-life–oriented strategies^50^. Individualized risk estimates could also inform optimized follow-up imaging schedules. For example, high-risk patients may require more frequent surveillance, whereas low-risk patients might benefit from extended imaging intervals, thereby reducing healthcare burden and gadolinium deposition^9,51^.

Several limitations of this study should be acknowledged. First, we used only conventional MRI sequences to maintain inter-site compatibility, excluding advanced functional modalities such as diffusion-weighted and perfusion imaging, which can capture tumor cellularity and vascular heterogeneity^52,53^. Future work should evaluate the added prognostic value of these modalities. Second, our analysis was limited to pre-operative data and therefore did not include post-operative and longitudinal variables—such as follow-up imaging, adjuvant treatment response, recurrence patterns, and evolving clinical status—that are known to influence long-term outcomes^54^. Future models should incorporate these factors, potentially as time-dependent covariates to avoid immortal-time bias, thereby enabling dynamic survival prediction. Third, the retrospective multicenter design posed challenges in data completeness and consistency. Particularly, key clinical, genetic, and treatment variables were frequently missing in the external validation cohorts, limiting comprehensive integration of prognostic factors. Prospective studies with standardized data collection and uniform imaging protocols are needed to validate GlioSurv’s utility in real-world, multi-institutional contexts.

In conclusion, GlioSurv is a multimodal transformer model employing an accelerated failure time (AFT) framework to integrate multiparametric MRI with clinical, molecular, and treatment data, providing precise and interpretable survival predictions for adult-type diffuse glioma. Across four independent validation cohorts, GlioSurv demonstrated robust performance and transparent identification of key prognostic variables. Personalized survival trajectories generated by GlioSurv may potentially inform tailored treatment planning, optimize follow-up imaging schedules, and support informed prognostic discussions within routine neuro-oncology practice.

## Methods

### Patient population

Between February 2008 and February 2024, we assessed 1,078 adult-type diffuse glioma patients treated at our internal institution. Inclusion criteria were: (1) histopathologically confirmed diagnosis of adult-type diffuse glioma^3^; (2) documented survival duration after diagnosis; and (3) age ≥ 18 years. We excluded 187 patients for several reasons: diffuse midline glioma, H3K27M-positive^55^ (*n* = 45); isocitrate dehydrogenase-wildtype (IDH-wt) diffuse glioma not otherwise specified (NOS)^56,57^ (*n* = 65); IDH-wt diffuse glioma not elsewhere classified (NEC)^57^ (*n* = 27); and incomplete magnetic resonance imaging (MRI) data (*n* = 50). The remaining 891 patients were randomly assigned in an 80:20 ratio to internal development (*n* = 713) and internal validation (*n* = 178) cohorts.

**Fig. 5:**
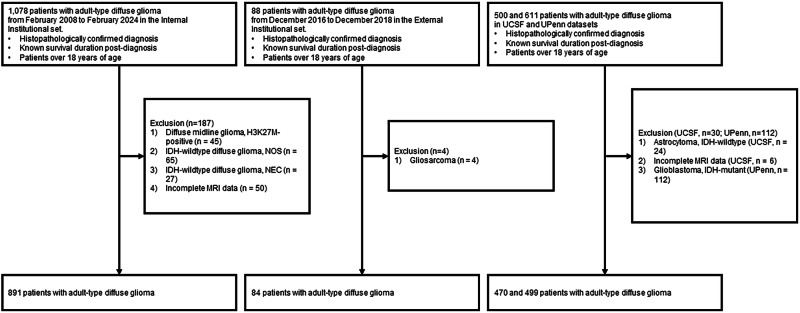
Flowchart illustrating patient cohort selection. Diagram detailing the screening, eligibility assessment, and final inclusion of patients from the four study cohorts: Internal Institutional (*n* = 891 initial, *n* = 713 development, *n* = 178 validation), External Institutional (*n* = 88 initial, *n* = 84 final), University of California San Francisco (UCSF) (*n* = 500 initial, *n* = 470 final), and University of Pennsylvania (UPenn) (*n* = 611 initial, *n* = 499 final). Numbers indicate patients at each stage.

For external validation, we initially assessed 88 adult-type diffuse glioma cases accrued between December 2016 and December 2018 at an external institution, applying identical inclusion and exclusion criteria. Four gliosarcoma variants were excluded, resulting in 84 evaluable patients. Subsequently, we applied the same selection criteria to publicly available cohorts from UCSF^33^ (*n* = 500) and UPenn^34^ (*n* = 611). From the UCSF set, 24 IDH-wt astrocytoma cases and six patients with incomplete MRI data were excluded, yielding 470 evaluable cases. From the UPenn set, 112 IDH-mutant glioblastoma patients were excluded, leaving 499 evaluable cases. The final study population therefore consisted of 713, 178, 84, 470, and 499 patients in the internal development, internal validation, external institutional, UCSF, and UPenn cohorts, respectively (Fig. 5).

### MRI acquisition parameters

Retrospective MRI datasets were gathered for all four cohorts. In the internal institutional cohort, examinations were conducted on 1.5–3 Tesla (T) scanners (Siemens, Philips, and GE) using 8–64-channel head coils. Protocols included volumetric three-dimensional T1-weighted sequences (repetition time [TR] = 8.4–1600 ms; echo time [TE] = 1.9–11 ms; field of view [FOV] = 220 × 220 to 256 × 256 mm; section thickness = 1 mm and 5 mm; matrix = 240 × 240 to 256 × 248). These were acquired before and after contrast administration (gadoterate meglumine, 0.1 mmol/kg; post-contrast parameters were identical, with a fixed 1 mm thickness). Conventional T2-weighted images utilized TR/TE = 4850–5100 ms/89–131 ms (FOV = 185 × 220 to 220 × 220 mm; thickness = 5 mm; matrix = 448 × 256 to 640 × 384). T2 fluid-attenuated inversion recovery (FLAIR) used TR/TE = 8700–9000 ms/90–100 ms, an inversion time of 2500 ms, FOV = 220 × 220 mm, thickness = 4 mm, and matrix = 512 × 512.

For the external institutional cohort, all scans were acquired on a single 3 T system (Siemens) with an 8-channel head coil. Three-dimensional T1-weighted imaging used TR/TE = 2000/10 ms, FOV = 240 × 240 mm, thickness = 5 mm, and a matrix of 256 × 256. Post-contrast T1 imaging used gadobutrol (0.1 mL/kg) with TR/TE = 9.8/4.6 ms, FOV = 240 × 240 mm, thickness = 1 mm, and a matrix of 224 × 224. T2-weighted images were acquired with TR/TE = 3000/80 ms, FOV = 240 × 240 mm, thickness = 5 mm, and a matrix of 256 × 256. T2 FLAIR employed TR/TE = 10000/125 ms, an inversion time of 2500 ms, FOV = 240 × 240 mm, thickness = 5 mm, and a matrix of 256 × 256. Protocol details for the UCSF and UPenn cohorts are described in their respective publications^33,34^.

### MRI preprocessing

All MRI scans underwent a consistent preprocessing pipeline. First, pre- and post-contrast T1-weighted, T2-weighted, and T2-FLAIR volumes were rigidly co-registered to the post-contrast T1-weighted (T1C) image using Advanced Normalization Tools (ANTs)^58^. Brain extraction was then performed on the registered T1C volume with SynthStrip^59^. Automated tumor segmentation used the HD-GLIO algorithm^60^ on skull-stripped images, yielding precise lesion masks. All sequences were subsequently resampled to a 1 × 1 × 4 mm³ anisotropic voxel grid and spatially standardized to 160 × 192 × 40 voxels by zero-padding or cropping. Finally, image intensities for each modality were normalized to zero mean and unit variance (z-score normalization) for each individual scan.

### Clinical and molecular data preparation

Clinical and molecular covariates were systematically gathered from electronic health records and pathology reports for all 1944 subjects. Any missing values were assumed to be missing at random (MAR). The proportion of missing data for each variable varied across the cohorts, as detailed in Supplementary Table 15. Demographic and performance data included age, sex, and KPS. Molecular and histopathological variables encompassed WHO tumor grade, histological subtype, IDH mutation status, 1p/19q codeletion, and MGMTp methylation. Treatment information detailed EOR, radiotherapy, and chemotherapy regimens. Overall survival was calculated from the date of diagnosis. To interface non-imaging data with the transformer architecture, each variable was transformed into a concise natural-language prompt (e.g., “The patient is male”; “The tumor is WHO grade IV”; “The patient underwent gross total resection”). Continuous metrics with known non-linear associations—specifically, age and KPS—were discretized into three categories (age ≤ 47, 48–63, ≥64 years; KPS ≤ 50, 60–70, ≥80)^61,62^. This discretization aimed to standardize input distributions and facilitate robust text encoder embedding.

### GlioSurv: multimodal transformer-based survival prediction model

**Fig. 6:**
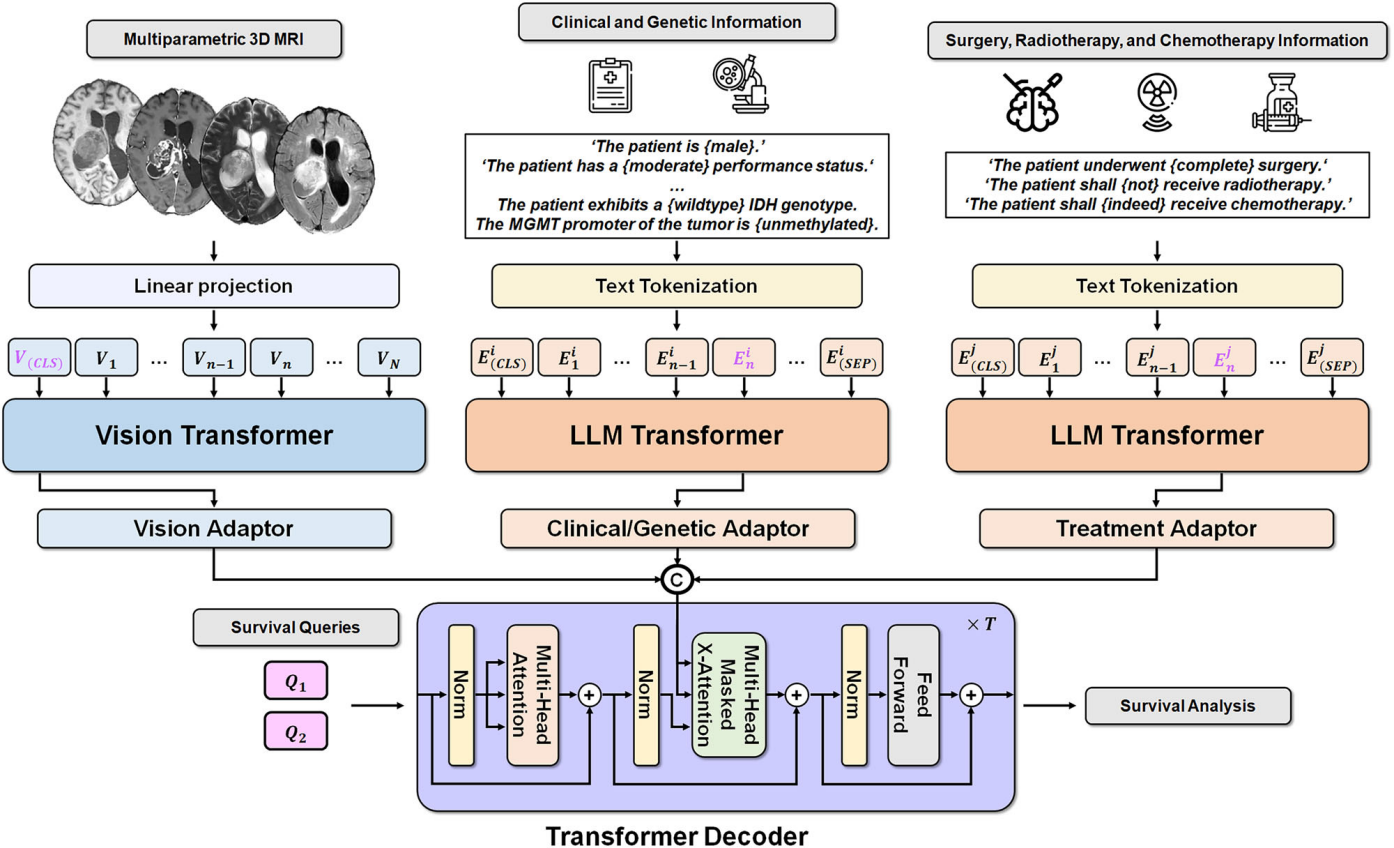
Architecture of the GlioSurv multimodal transformer model. A schematic representation of the GlioSurv framework. Three parallel streams process (left to right): (1) multiparametric magnetic resonance imaging (MRI) data, (2) clinical and genetic variables, and (3) treatment information. MRI volumes undergo patch embedding, are encoded by a vision transformer (ViT), and then refined by a Vision Adaptor. Clinical/genetic variables and treatment data are converted into natural language text prompts, encoded using a Bidirectional Encoder Representations from Transformers (BERT) model, and subsequently refined by modality-specific adaptors (Clinical/Genetic Adaptor and Treatment Adaptor). These modality-specific representations converge in a central fusion module, where learnable Survival Query tokens integrate information across modalities via masked cross-attention mechanisms. The aggregated representation is then passed to an accelerated failure time (AFT) model head, which directly estimates patient-specific survival distribution parameters.

GlioSurv is a unified transformer–based framework that integrates multiparametric MRI, structured clinical/genetic covariates, and treatment information for individualized diffuse glioma survival prediction (Fig. 6). For the imaging branch, a learnable linear embedding layer projected 3D MRI volumes into a sequence of fixed-length feature vectors. A Vision Transformer (ViT)^63^ processed these embeddings, followed by a two-layer, modality-specific adaptor (Vision Adaptor) that refined visual representations for survival prediction^64^.

Clinical and molecular variables (age, sex, KPS, WHO grade, IDH mutation, 1p/19q codeletion, MGMTp methylation) were converted to natural-language prompts, then tokenized, embedded, and encoded by a pretrained Bidirectional Encoder Representations from Transformers (BERT) model^65^. Subsequently, a two-layer modality-specific adaptor (Clinical/Genetic Adaptor) transformed these embeddings into feature vectors optimized for multimodal fusion. Treatment variables—including EOR, radiotherapy, and chemotherapy—were processed using a parallel BERT-based branch equipped with a modality-specific adaptor (Treatment Adaptor).

Outputs from the imaging, clinical/genetic, and treatment branches were combined in a cross-attention–driven fusion module. Here, learnable “Survival Query” tokens interact with modality-specific embeddings via multi-head and masked cross-attention layers. This approach effectively addresses missing inputs and enhances robustness to incomplete records. The aggregated multimodal representation feeds into an AFT-based survival head, which is trained by minimizing the negative log-likelihood, to directly estimate patient-specific time-to-event distributions. This end-to-end architecture allows GlioSurv to learn complex interdependencies across heterogeneous data, thereby yielding precise, interpretable survival predictions without requiring subsequent calibration.

### Model comparison

We implemented three deep learning architectures to compare distinct architectural and modality-driven strategies for survival prediction. A 3D convolutional neural network (CNN) was implemented using a ResNeXt backbone^66^. Multiparametric MRI volumes were processed via grouped convolutions and residual connections to extract spatial features. These were passed through fully connected layers to generate individualized survival estimates.

A Vision Transformer (ViT)^63^ was extended to handle three-dimensional inputs. MRI volumes were divided into non-overlapping 3D patches, each linearly embedded with positional encodings. The patch sequence was processed by a transformer encoder, and a learnable classification token aggregated global contextual information for downstream prediction.

To isolate the prognostic contribution of imaging, we designed the Non-Imaging Multimodal Transformer (NIMT), an ablated GlioSurv variant without an imaging encoder. This model relies exclusively on clinical, molecular, and treatment variables, reformulated as natural language prompts and encoded using a pretrained BERT model—the same method employed in GlioSurv. NIMT retains the transformer-based fusion module and prediction head, enabling a controlled assessment of imaging’s added value.

To validate the model’s built-in method for handling missing data, we compared the performance of GlioSurv’s masked cross-attention mechanism against two conventional imputation strategies: mode imputation and multivariate imputation by chained equations (MICE)^67^. The masked cross-attention approach demonstrated superior IAUC, IBS, and C-index, outperforming both imputation methods with statistical significance in the majority of comparisons across the validation cohorts (Supplementary Table 16). All models were trained using identical preprocessing and optimization pipelines to ensure consistency across evaluations.

### Model training and survival prediction strategy

We adopted a two-stage training approach to mitigate cohort-specific MRI acquisition variability (e.g., scanner vendor, field strength, coil configuration, protocol differences)^68^. In the first stage, the ViT was pretrained exclusively on multiparametric MRI volumes to learn robust imaging representations. This pretraining was performed exclusively on the multiparametric MRI volumes from the internal development cohort (*n* = 713), ensuring that no data from the internal validation or any external validation cohorts were used, thereby preventing data leakage. After pretraining, the ViT weights were frozen. The BERT language model, used for encoding clinical, genetic, and treatment prompts, was also kept frozen throughout training to prevent overfitting and minimize computational cost.

In the second stage, the frozen ViT and BERT encoders provided input to the GlioSurv fusion architecture. Learnable Survival Query tokens (Q₁ and Q₂) attend to multimodal embeddings via cross-attention. These tokens parameterize the log-scale (log λ) and log-shape (log ρ) of a Weibull AFT model^32^, enabling direct estimation of individualized time-to-event distributions. Model parameters were optimized by minimizing the negative log-likelihood (NLL) of observed survival times and censoring indicators.

The model was trained for a maximum of 1000 epochs, with an early stopping criterion based on the C-index of the validation set. To enhance model robustness, we applied a series of data augmentations during training, including random flipping, random affine transformations (rotation, translation, and scaling), and random contrast adjustments. Training and evaluation were conducted using PyTorch 2.1.2 on NVIDIA RTX A6000 GPUs with Python 3.10. For comprehensive implementation details, including library versions and random seeds, please refer to our public code repository: [https://github.com/snuh-rad-aicon/GlioSurv.git].

### Interpretability analysis methods

We applied two complementary techniques to clarify GlioSurv’s decision-making process and visualize the spatial basis of its survival estimates. First, we generated activation maps from the ViT branch using Eigen-Class Activation Mapping (Eigen-CAM)^45^. Eigen-CAM employs principal component analysis on feature maps to highlight tumor regions that strongly influence model output. Second, we quantified prognostic variable contributions—encompassing imaging-derived features, clinical/genetic covariates, and treatment details—by computing relevance scores with Gradient-weighted Class Activation Mapping (Grad-CAM)^46^. In addition, we employed SHapley Additive exPlanations (SHAP)^35^, a game theory-based method, to provide a robust quantification of each feature’s contribution to individual predictions. Positive relevance values denote factors increasing predicted risk, while negative values indicate protective effects. Both Eigen-CAM and Grad-CAM analyses, along with SHAP analyses, were performed on individual patient cases, stratified by histopathological subtype, MGMTp methylation status, and EOR. This provided patient-specific insights into the features underlying GlioSurv’s predictions.

### Predicting individual survival probabilities

Individualized survival probabilities were derived using patient-specific log-scale (log λ) and log-shape (log ρ) parameters obtained from GlioSurv’s AFT head. The selection of the Weibull distribution for the AFT model was based on a goodness-of-fit analysis that compared five different parametric survival models. This analysis confirmed that the Weibull model consistently provided one of the best fits to the observed survival data across all cohorts when benchmarked against the non-parametric Kaplan-Meier estimate (Supplementary Fig. 7).

For each patient, their Weibull survival function, $S\left(t\right)={e}^{-{\left(\frac{t}{\lambda }\right)}^{\rho }}$, was evaluated at discrete timepoints to generate personalized survival curves. To quantify uncertainty, we performed Monte Carlo sampling (10,000 draws) from the parametric Weibull distribution (parameterized by the estimated λ and ρ). The upper and lower 2.5th percentiles were excluded to derive 95% confidence intervals. Resulting samples were then smoothed using Kernel Density Estimation (KDE) to produce continuous density curves, which illustrate the probabilistic distribution of predicted survival times. Overlaying these density estimates and survival curves with observed outcomes for selected cases demonstrated GlioSurv’s capacity for precise, interpretable, and uncertainty-aware prognostic insights at the individual patient level.

### Statistical analysis

Continuous variables are presented as mean ± standard deviation (or median [interquartile range]), and categorical variables are presented as n (%). Between-group comparisons utilized chi-square tests for categorical variables and one-way analysis of variance (ANOVA) for continuous variables. Model discrimination was quantified by C-index and time-dependent AUC, with the integrated AUC (IAUC) derived from trapezoidal integration. Calibration was assessed via time-dependent Brier score, with the integrated Brier score (IBS) computed similarly. Additionally, calibration slope and intercept at 6 and 12 months were estimated via logistic regression of observed outcomes on predicted risk, and decision curve analysis was used to quantify the net benefit across a range of threshold probabilities. Two-sided 95% confidence intervals were generated by nonparametric percentile bootstrap (*n* = 999). Hypothesis testing employed Wald-type tests for regression coefficients and permutation-based bootstrap tests for model comparisons. Pairwise comparisons of IAUC, IBS, and C-index between models were performed using paired bootstrap tests. For subgroup analyses, model performance was compared using two-sample bootstrap tests. All model comparisons were considered exploratory, and consequently, no adjustments for multiple comparisons were applied. Kaplan–Meier survival curves were estimated and subsequently compared using the log-rank test. Statistical significance was set at a two-sided *P* < 0.05. All analyses were conducted in Python (SciPy^69^, lifelines^70^, scikit-learn^71^, and torchsurv^72^).

### Ethical approval

This retrospective, multicenter cohort study utilized de-identified clinical, molecular, and imaging data from four tertiary neuro-oncology centers. The study conformed to the principles of the 1975 Declaration of Helsinki and its subsequent revisions. Institutional Review Board (IRB) approval was obtained at each participating site (IRB No. XXXX). A waiver of written informed consent was granted due to the retrospective and anonymized nature of the data.

## Supplementary information

Supplementary information

## Data Availability

The datasets generated and analyzed during this study are available from the corresponding author upon reasonable request, subject to institutional data sharing agreements. The UCSF and UPenn datasets are publicly available at [https://www.cancerimagingarchive.net/collection/ucsf-pdgm/] and [https://www.cancerimagingarchive.net/collection/upenn-gbm/]. The code for the GlioSurv model and analysis pipeline is available at [https://github.com/snuh-rad-aicon/GlioSurv.git].
