# Supplementary material for: GlioSurv: interpretable transformer for multimodal, individualized survival prediction in diffuse glioma
Source: NPJ Digit Med. 2025 Nov 14;8:660. doi: 10.1038/s41746-025-02018-x (PMC12618496; doi:10.1038/s41746-025-02018-x)
Supplement: Supplementary file 1 — Supplementary information [file 41746_2025_2018_MOESM1_ESM.pdf]

Supplementary Information

GlioSurv: Interpretable Transformer for Multimodal, Individualized Survival Prediction in Diffuse Glioma

Supplementary Figure 1. Kaplan-Meier survival analysis stratified by key clinical and molecular prognostic factors in the combined cohort.

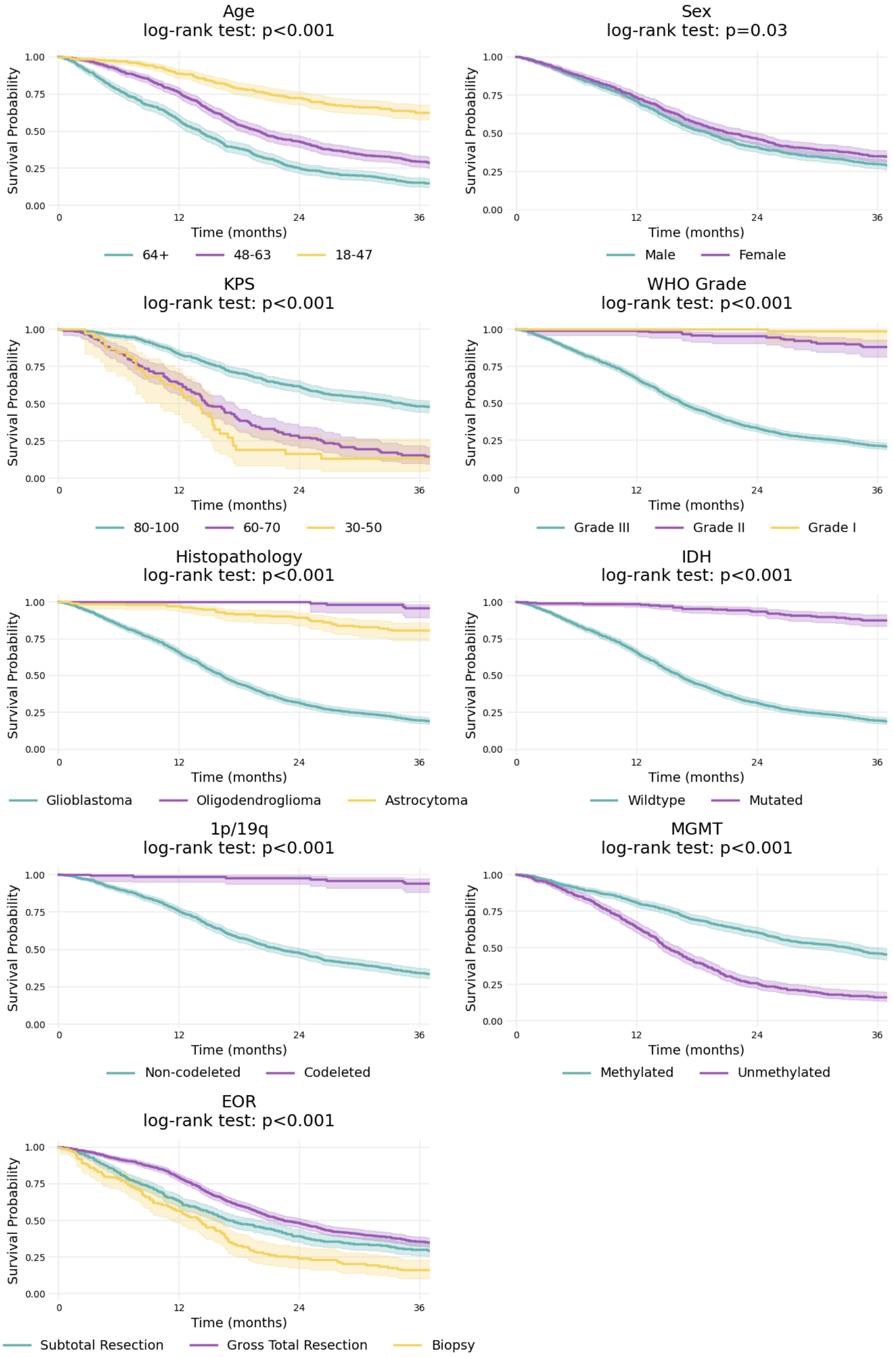

Kaplan–Meier curves illustrating overall survival probabilities for patient subgroups defined by established prognostic variables. Factors include: (a) age at diagnosis (18–47 years, 48–63 years, ≥64 years), (b) sex (male vs. female), (c) Karnofsky Performance Status (KPS; e.g., ≤70 vs. >70, or other relevant clinical cutoffs), (d) World Health Organization (WHO) grade (e.g., Grade IV vs. Grade II/III), (e) histopathological subtype (glioblastoma, isocitrate dehydrogenase [IDH]-wildtype; astrocytoma, IDH-mutant; oligodendroglioma, IDH-mutant and 1p/19q-codeleted), (f) IDH mutation status (mutant vs. wildtype), (g) 1p/19q codeletion status (codeleted vs. non-codeleted), (h) O6-methylguanine-DNA methyltransferase promoter (MGMTp) methylation status (methylated vs. unmethylated), and (i) extent of resection (EOR; e.g., gross total resection vs. subtotal resection vs. biopsy). Shaded regions represent 95% confidence intervals. Log-rank P-values assess the statistical significance of survival differences between subgroups.

Supplementary Figure 2. Calibration Slope and Intercept Analysis.

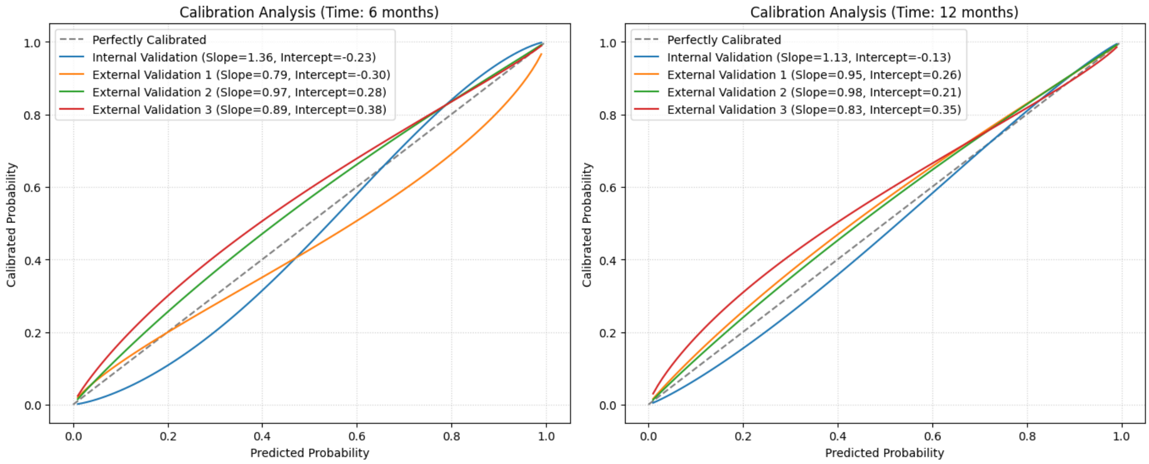

Calibration trendlines for the GlioSurv model across the internal and three external validation cohorts, evaluated at clinically relevant timepoints of (a) 6 months and (b) 12 months. The dashed diagonal line represents perfect calibration (Slope = 1.0, Intercept = 0.0). Solid lines depict the fitted calibration trend for each cohort, with corresponding calibration slope and intercept values reported in the legend.

Supplementary Figure 3. Risk stratification by CNN (imaging-only) versus NIMT (clinical-only) using different data modalities.

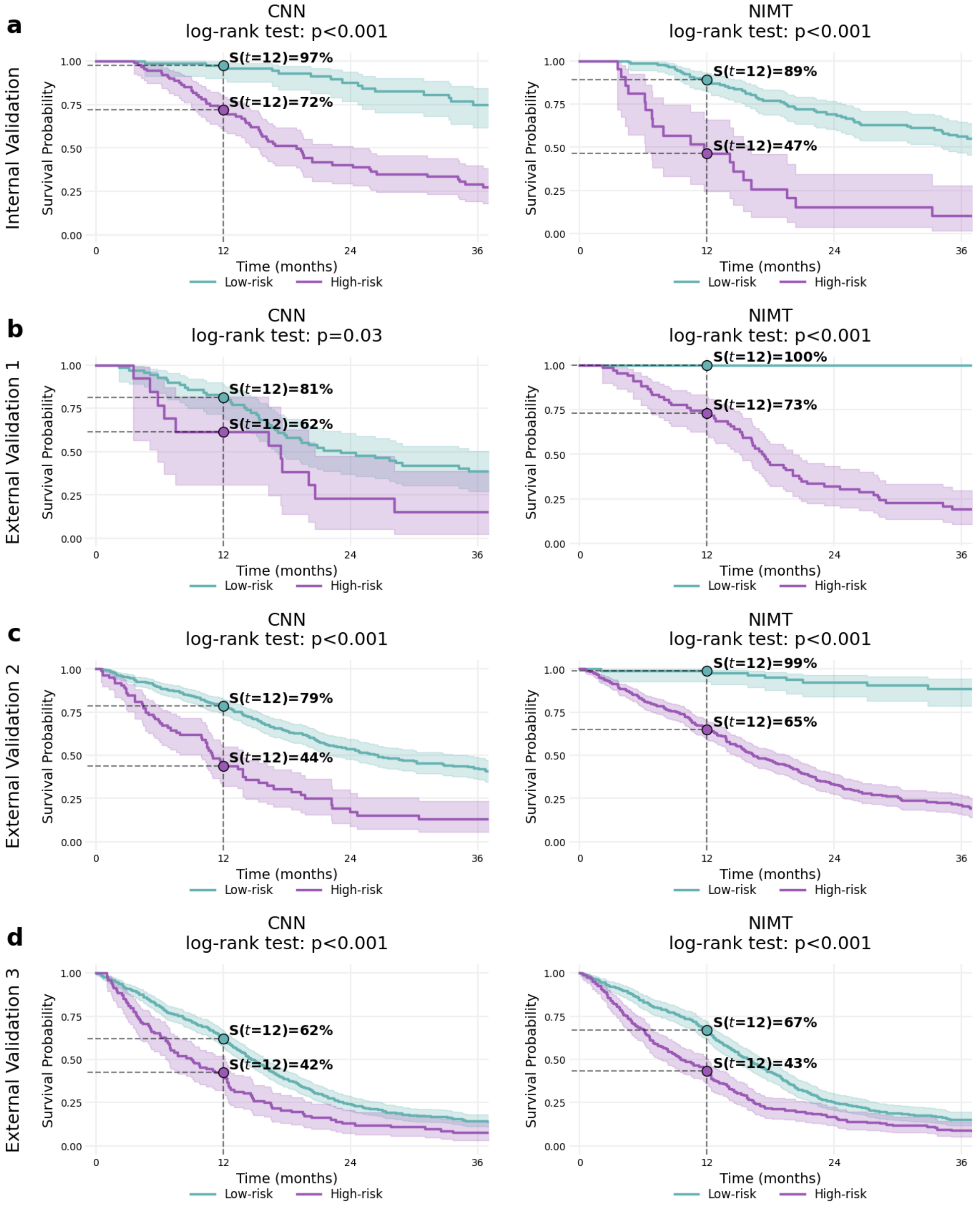

Kaplan–Meier estimates of overall survival for patients classified as low-risk (blue lines) and high-risk (red lines) based on the median predicted risk score from Convolutional Neural Network (CNN) and Non-Imaging Multimodal Transformer (NIMT). Survival curves compare CNN (left column) and NIMT (right column) predictions across: (a) internal institutional cohort, (b) external institutional cohort, (c) University of California San Francisco (UCSF) cohort, and (d) University of Pennsylvania (UPenn) cohort. Vertical dashed lines mark the 12-month timepoint, with corresponding survival probabilities annotated. Log-rank P-values assess the significance of separation between risk groups for each model in each cohort.

Supplementary Figure 4. Representative examples of accurate tumor localization using Eigen-CAM across different validation cohorts.

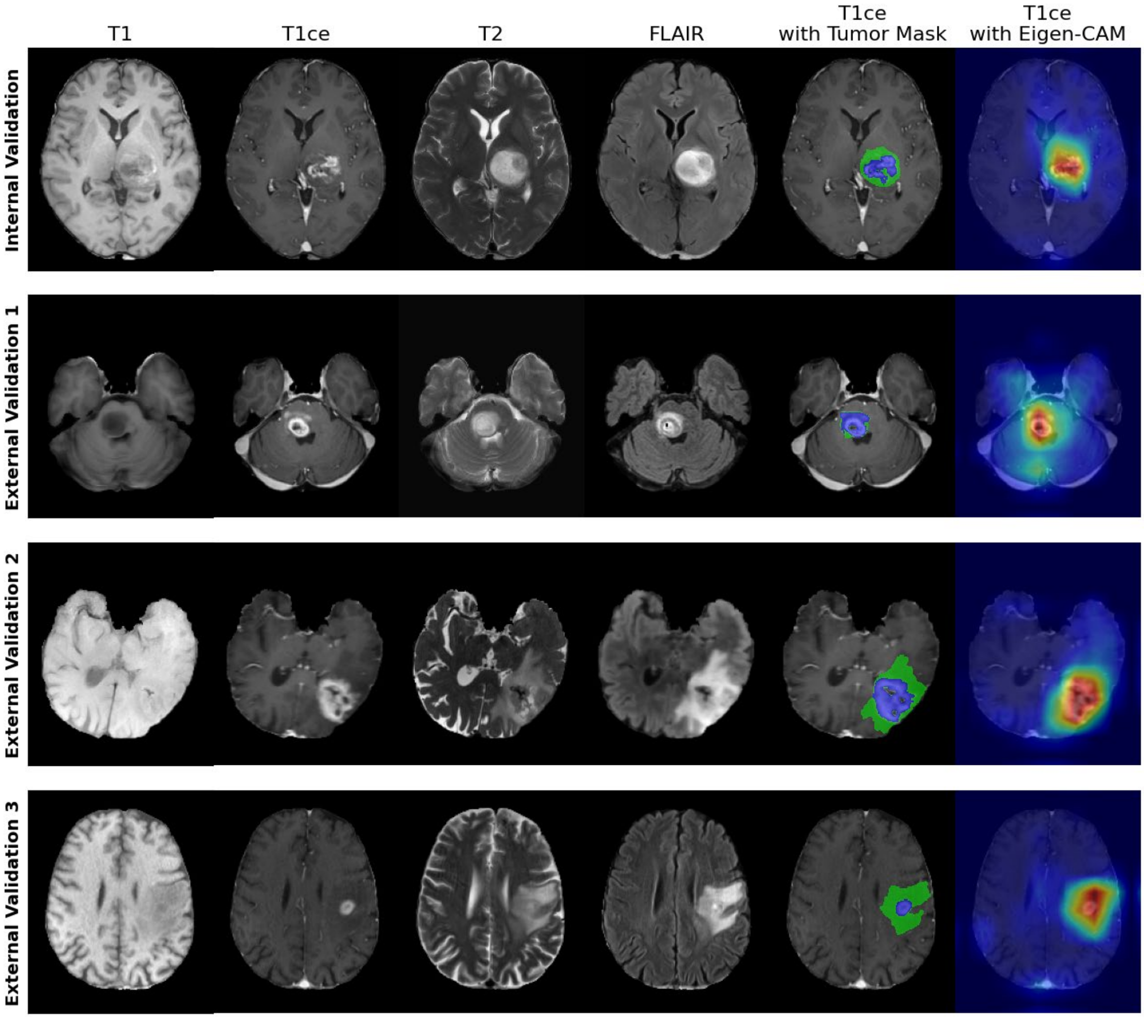

Qualitative examples illustrating accurate tumor localization by the model's Eigen-Class Activation Mapping (Eigen-CAM) across the four validation cohorts. Each row displays a representative patient from the internal validation, external validation 1, external validation 2, and external validation 3 cohorts. Columns show (from left to right): T1-weighted, T1-contrast enhanced (T1ce), T2-weighted, and Fluid-Attenuated Inversion Recovery (FLAIR) magnetic resonance imaging sequences. The final two columns overlay the ground-truth tumor segmentation mask and the Eigen-CAM activation map, respectively, onto the T1ce image.

Supplementary Figure 5. Representative examples of inaccurate tumor localization using Eigen-CAM across different validation cohorts.

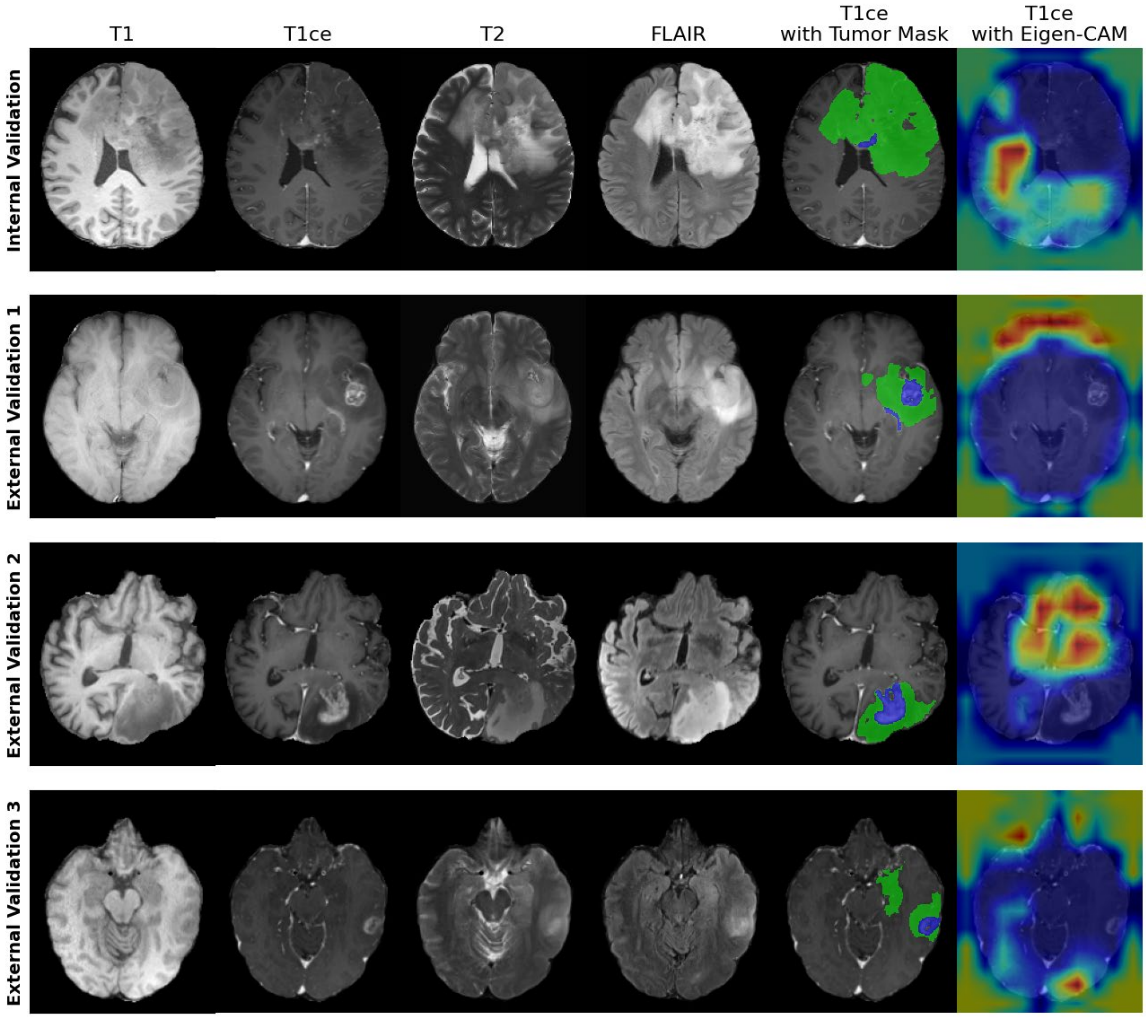

Qualitative examples illustrating accurate tumor localization by the model's Eigen-Class Activation Mapping (Eigen-CAM) across the four validation cohorts. Each row displays a representative patient from the internal validation, external validation 1, external validation 2, and external validation 3 cohorts. Columns show (from left to right): T1-weighted, T1-contrast enhanced (T1ce), T2-weighted, and Fluid-Attenuated Inversion Recovery (FLAIR) magnetic resonance imaging sequences. The final two columns overlay the ground-truth tumor segmentation mask and the Eigen-CAM activation map, respectively, onto the T1ce image.

Supplementary Figure 6. Decision Curve Analysis of GlioSurv at 12 Months.

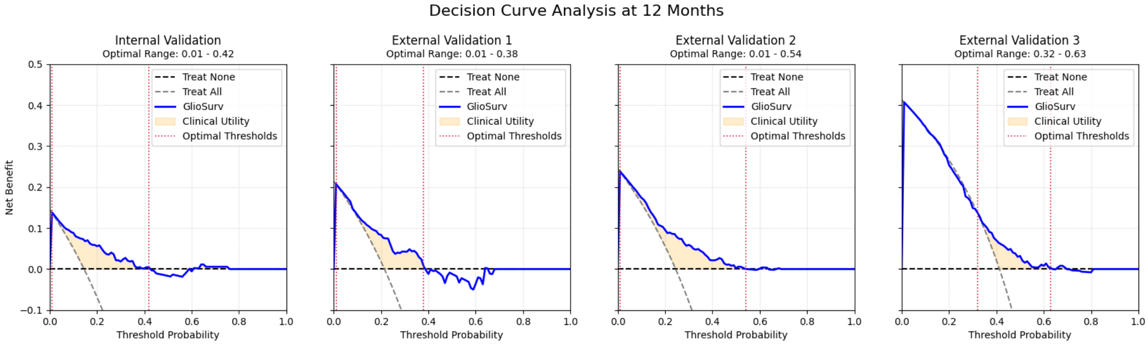

Decision curve analysis (DCA) of the GlioSurv model for 12-month mortality prediction across four cohorts: (a) Internal Validation, (b) External Validation 1, (c) External Validation 2, and (d) External Validation 3. Each curve plots the clinical net benefit over a range of threshold probabilities. The performance of the GlioSurv model (blue line) is compared against two default strategies: treating all patients (gray dashed line) and treating no patients (black dashed line). The shaded orange area highlights the range of thresholds where the model demonstrates a superior net benefit over both default strategies, with vertical dotted lines indicating the optimal range.

Supplementary Figure 7. Goodness-of-Fit Assessment for Parametric Survival Models.

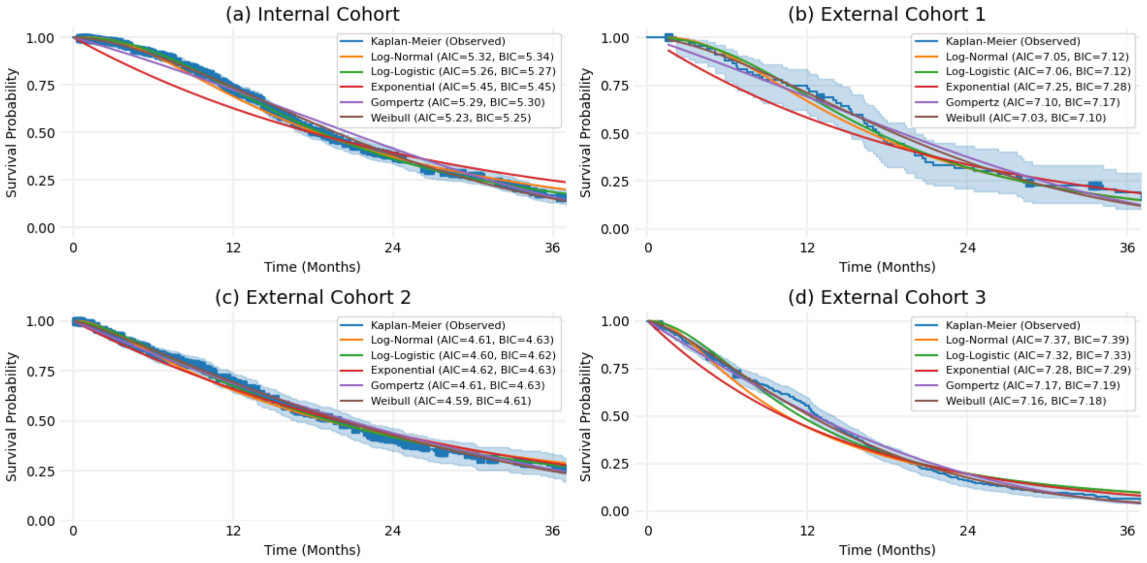

Goodness-of-fit comparison for five parametric survival models—Weibull, Log-Normal, Log-Logistic, Gompertz, and Exponential—across four cohorts: (a) Internal Cohort, (b) External Cohort 1, (c) External Cohort 2, and (d) External Cohort 3. In each panel, the fitted parametric curves are benchmarked against the non-parametric Kaplan-Meier (KM) survival estimate (black line), which represents the observed survival function. Shaded areas denote the 95% confidence interval for the KM estimate, and squares indicate censored events. The legend displays the normalized Akaike Information Criterion (AIC) and Bayesian Information Criterion (BIC) for each model, where lower values signify a better fit to the observed data.

Supplementary Table 1. Baseline clinicopathological characteristics of the internal development and validation sets

Characteristics	Internal Development set (n=712)	Internal Validation set (n=179)	P value
**Age**	55.3 ± 14.7	54.3 ± 14.0	0.422
**Sex**			0.403
**Male**	409/712 (57.4)	96/179 (53.6)	
**Female**	303/712 (42.6)	83/179 (46.4)	
**Karnofsky performance status**			0.209
**100**	233/712 (32.7)	53/179 (29.6)	
**90**	190/712 (26.7)	67/179 (37.4)	
**80**	102/712 (14.3)	19/179 (10.6)	
**70**	71/712 (10.0)	18/179 (10.1)	
**60**	38/712 (5.3)	5/179 (2.8)	
**50**	18/712 (2.5)	4/179 (2.2)	
**40**	4/712 (0.6)	0/179 (0.0)	
**30**	1/712 (0.1)	0/179 (0.0)	
**Unknown**	55/712 (7.7)	13/179 (7.3)	
**WHO Grade**			0.516
**Grade 2**	56/712 (7.9)	17/179 (9.5)	
**Grade 3**	106/712 (14.9)	31/179 (17.3)	
**Grade 4**	550/712 (77.2)	131/179 (73.2)	
**Histopathology**			0.530
**Glioblastoma, IDH wildtype**	515/712 (72.3)	123/179 (68.7)	
**Astrocytoma, IDH-mutant**	90/712 (12.6)	28/179 (15.6)	
**Oligodendroglioma, IDH-mutant, and 1p/19q-codeleted**	107/712 (15.0)	28/179 (15.6)	
**IDH mutation**			0.386
**Wildtype**	515/712 (72.3)	123/179 (68.7)	
**Mutated**	197/712 (27.7)	56/179 (31.3)	
**1p/19q co-deletion**			0.794
**Non-codeleted**	576/712 (80.9)	141/179 (78.8)	
**Codeleted**	113/712 (15.9)	31/179 (17.3)	
**Unknown**	23/712 (3.2)	7/179 (3.9)	
**MGMTp methylation**			0.179
**Unmethylated**	310/712 (43.5)	72/179 (40.2)	
**Methylated**	392/712 (55.1)	101/179 (56.4)	
**Unknown**	10/712 (1.4)	6/179 (3.4)	
**Extent of resection**			0.595
**Gross Total Resection**	440/712 (61.8)	111/179 (62.0)	
**Subtotal Resection**	177/712 (24.9)	44/179 (24.6)	
**Biopsy**	88/712 (12.4)	24/179 (13.4)	
**Unknown**	7/712 (1.0)	0/179 (0.0)	
**Radiation therapy**			0.104
**Received**	520/712 (73.0)	142/179 (79.3)	
**Not Received**	192/712 (27.0)	37/179 (20.7)	
**Unknown**	0/712 (0.0)	0/179 (0.0)	
**Chemotherapy**			0.388
**Received**	438/712 (61.5)	117/179 (65.4)	
**Not Received**	274/712 (38.5)	62/179 (34.6)	
**Unknown**	0/712 (0.0)	0/179 (0.0)	
**Overall survival (Months)**	31.1 ± 34.2	36.6 ± 39.4	0.066
**Death**			0.254
**Occurred**	414/712 (58.1)	95/179 (53.1)	
**Censored**	298/712 (41.9)	84/179 (46.9)	

Continuous variables are presented as mean ± standard deviation, and categorical variables as n (%). P-values were calculated using one-way analysis of variance for continuous variables and chi-square test for categorical variables. Overall survival was compared using a multivariate log-rank test.

Abbreviations: GTR, gross total resection; IDH, isocitrate dehydrogenase; MGMTp, O6-methylguanine-DNA methyltransferase promoter; UCSF, University of California, San Francisco; UPenn, University of Pennsylvania; WHO, World Health Organization.

Supplementary Table 2. Subgroup analysis based on data completeness across validation cohorts.

Center	Metric	(1) Subgroup with Complete Data	(2) Subgroup with Incomplete Data	P value (1) vs (2)
**Internal Validation**	IAUC	0.87 [0.83, 0.91]	0.68 [0.47, 0.99]	<0.001
	IBS	0.10 [0.08, 0.11]	0.18 [0.13, 0.24]	<0.001
	C-index	0.81 [0.76, 0.85]	0.70 [0.36, 0.96]	<0.001
**External Validation 1**	IAUC	0.75 [0.64, 0.83]	N/A	N/A
	IBS	0.17 [0.14, 0.21]	N/A	N/A
	C-index	0.70 [0.62, 0.77]	N/A	N/A
**External Validation 2**	IAUC	0.79 [0.75, 0.84]	0.76 [0.70, 0.82]	<0.001
	IBS	0.14 [0.12, 0.16]	0.14 [0.12, 0.17]	<0.001
	C-index	0.74 [0.69, 0.78]	0.67 [0.61, 0.73]	<0.001
**External Validation 3**	IAUC	0.73 [0.69, 0.79]	0.62 [0.57, 0.68]	<0.001
	IBS	0.20 [0.18, 0.21]	0.22 [0.21, 0.24]	<0.001
	C-index	0.65 [0.62, 0.69]	0.58 [0.54, 0.62]	<0.001

The dataset was partitioned into two mutually exclusive groups: (1) the 'Complete Data Subgroup', consisting of all patients with no missing values for any of the specified variables (WHO Grade, Histopathology, IDH mutation, 1p/19q co-deletion, MGMTp methylation, Extent of resection), and (2) the 'Incomplete Data Subgroup', consisting of all patients with at least one missing value in this set. Values are presented as metric [95% confidence interval]. P-values were calculated using two-sample bootstrap tests. N/A (not applicable) denotes instances where the metric could not be robustly estimated via bootstrapping, typically due to an insufficient number of events within the subgroup.

Abbreviations: C-index, concordance index; IAUC, integrated area under the time-dependent receiver operating characteristic curve; IBS, integrated Brier score.

Supplementary Table 3. Subgroup analysis based on Age across validation cohorts.

Center	Metric	(1) 15-47	(2) 48-63	(3) 64+	P value (1) vs (2)	P value (1) vs (3)	P value (2) vs (3)
**Internal Validation**	IAUC	0.92 [0.25, 0.54]	0.84 [0.41, 0.67]	0.85 [0.49, 0.83]	0.120	0.026	0.236
	IBS	0.08 [0.13, 0.27]	0.12 [0.13, 0.21]	0.10 [0.10, 0.20]	0.574	0.314	0.482
	C-index	0.87 [0.80, 0.93]	0.74 [0.65, 0.81]	0.80 [0.67, 0.86]	0.008	0.136	0.516
**External Validation 1**	IAUC	0.90 [0.49, 0.85]	0.71 [0.17, 0.50]	0.55 [0.26, 0.60]	0.002	0.058	0.474
	IBS	0.10 [0.10, 0.26]	0.18 [0.22, 0.36]	0.21 [0.20, 0.32]	0.046	0.112	0.468
	C-index	0.89 [0.75, 1.00]	0.67 [0.49, 0.79]	0.60 [0.43, 0.70]	0.026	<0.001	0.372
**External Validation 2**	IAUC	0.87 [0.30, 0.53]	0.70 [0.36, 0.54]	0.66 [0.36, 0.54]	0.636	0.642	0.896
	IBS	0.07 [0.08, 0.15]	0.16 [0.18, 0.24]	0.18 [0.19, 0.25]	<0.001	<0.001	0.514
	C-index	0.82 [0.74, 0.87]	0.65 [0.57, 0.70]	0.61 [0.54, 0.67]	0.004	<0.001	0.376
**External Validation 3**	IAUC	0.61 [0.33, 0.64]	0.68 [0.43, 0.55]	0.66 [0.44, 0.57]	0.964	0.852	0.696
	IBS	0.23 [0.21, 0.31]	0.20 [0.23, 0.26]	0.21 [0.23, 0.26]	0.538	0.532	0.852
	C-index	0.58 [0.46, 0.67]	0.62 [0.57, 0.65]	0.60 [0.55, 0.62]	0.516	0.846	0.438

Values are presented as metric [95% confidence interval]. P-values were calculated using two-sample bootstrap tests. N/A (not applicable) denotes instances where the metric could not be robustly estimated via bootstrapping, typically due to an insufficient number of events within the subgroup.

Abbreviations: C-index, concordance index; IAUC, integrated area under the time-dependent receiver operating characteristic curve; IBS, integrated Brier score.

Supplementary Table 4. Subgroup analysis based on Karnofsky performance status across validation cohorts.

Center	Metric	(1) 30-50	(2) 60-70	(3) 80-100	P value (1) vs (2)	P value (1) vs (3)	P value (2) vs (3)
**Internal Validation**	IAUC	0.39 [0.00, 0.00]	0.73 [0.45, 0.92]	0.89 [0.30, 0.48]	<0.001	<0.001	0.014
	IBS	0.25 [0.06, 0.58]	0.14 [0.10, 0.23]	0.09 [0.13, 0.21]	0.370	0.166	0.606
	C-index	N/A	0.70 [0.47, 0.83]	0.82 [0.77, 0.86]	N/A	N/A	0.078
**External Validation 1**	IAUC	0.39 [0.28, 0.95]	0.66 [0.29, 0.63]	0.79 [0.35, 0.63]	0.434	0.452	0.588
	IBS	0.20 [0.13, 0.32]	0.20 [0.19, 0.32]	0.15 [0.22, 0.35]	0.442	0.230	0.712
	C-index	0.58 [0.23, 0.79]	0.60 [0.47, 0.73]	0.75 [0.66, 0.86]	0.726	0.112	0.054
**External Validation 2**	IAUC	N/A	N/A	N/A	N/A	N/A	N/A
	IBS	N/A	N/A	N/A	N/A	N/A	N/A
	C-index	N/A	N/A	N/A	N/A	N/A	N/A
**External Validation 3**	IAUC	0.15 [0.00, 0.70]	0.63 [0.41, 0.88]	0.77 [0.44, 0.71]	0.428	0.706	0.588
	IBS	0.39 [0.13, 0.61]	0.26 [0.15, 0.39]	0.21 [0.21, 0.28]	0.284	0.324	0.970
	C-index	N/A	0.60 [0.36, 0.92]	0.67 [0.60, 0.76]	N/A	N/A	0.942

Values are presented as metric [95% confidence interval]. P-values were calculated using two-sample bootstrap tests. N/A (not applicable) denotes instances where the metric could not be robustly estimated via bootstrapping, typically due to an insufficient number of events within the subgroup.

Abbreviations: C-index, concordance index; IAUC, integrated area under the time-dependent receiver operating characteristic curve; IBS, integrated Brier score.

Supplementary Table 5. Subgroup analysis based on WHO Grade across validation cohorts.

Center	Metric	(1) Grade 2	(2) Grade 3	(3) Grade 4	P value (1) vs (2)	P value (1) vs (3)	P value (2) vs (3)
**Internal Validation**	IAUC	N/A	N/A	0.80 [0.47, 0.67]	N/A	N/A	N/A
	IBS	0.02 [0.00, 0.07]	0.01 [0.00, 0.01]	0.13 [0.15, 0.21]	0.540	<0.001	<0.001
	C-index	N/A	0.74 [0.39, 0.94]	0.71 [0.64, 0.75]	N/A	N/A	0.806
**External Validation 1**	IAUC	N/A	0.82 [0.52, 0.95]	0.64 [0.33, 0.56]	N/A	N/A	0.012
	IBS	N/A	0.13 [0.10, 0.23]	0.20 [0.22, 0.30]	N/A	N/A	0.006
	C-index	N/A	0.74 [0.50, 1.00]	0.60 [0.49, 0.68]	N/A	N/A	0.144
**External Validation 2**	IAUC	N/A	N/A	0.71 [0.41, 0.54]	N/A	N/A	N/A
	IBS	0.00 [0.00, 0.00]	0.04 [0.02, 0.08]	0.17 [0.19, 0.22]	<0.001	<0.001	<0.001
	C-index	N/A	0.65 [0.21, 1.00]	0.63 [0.60, 0.68]	N/A	N/A	0.912
**External Validation 3**	IAUC	N/A	N/A	0.68 [0.45, 0.55]	N/A	N/A	N/A
	IBS	N/A	N/A	0.21 [0.24, 0.26]	N/A	N/A	N/A
	C-index	N/A	N/A	0.61 [0.59, 0.64]	N/A	N/A	N/A

Values are presented as metric [95% confidence interval]. P-values were calculated using two-sample bootstrap tests. N/A (not applicable) denotes instances where the metric could not be robustly estimated via bootstrapping, typically due to an insufficient number of events within the subgroup.

Abbreviations: C-index, concordance index; IAUC, integrated area under the time-dependent receiver operating characteristic curve; IBS, integrated Brier score.

Supplementary Table 6. Subgroup analysis based on Histopathology across validation cohorts.

Center	Metric	(1) Glioblastoma	(2) Astrocytoma	(3) Oligodendroglioma	P value (1) vs (2)	P value (1) vs (3)	P value (2) vs (3)
**Internal Validation**	IAUC	0.80 [0.40, 0.61]	N/A	N/A	N/A	N/A	N/A
	IBS	0.14 [0.16, 0.23]	0.05 [0.02, 0.13]	0.01 [0.00, 0.03]	0.002	<0.001	0.012
	C-index	0.71 [0.64, 0.75]	0.80 [0.58, 0.96]	0.55 [0.21, 1.00]	0.250	0.600	0.390
**External Validation 1**	IAUC	0.64 [0.33, 0.57]	0.84 [0.57, 0.96]	N/A	0.008	N/A	N/A
	IBS	0.20 [0.22, 0.30]	0.12 [0.07, 0.21]	N/A	<0.001	N/A	N/A
	C-index	0.60 [0.50, 0.69]	0.76 [0.56, 1.00]	N/A	0.144	N/A	N/A
**External Validation 2**	IAUC	0.68 [0.42, 0.54]	0.86 [0.28, 0.54]	N/A	0.436	N/A	N/A
	IBS	0.17 [0.18, 0.22]	0.04 [0.02, 0.08]	N/A	<0.001	N/A	N/A
	C-index	0.62 [0.59, 0.67]	0.75 [0.63, 0.91]	N/A	0.054	N/A	N/A
**External Validation 3**	IAUC	0.68 [0.46, 0.54]	N/A	N/A	N/A	N/A	N/A
	IBS	0.21 [0.24, 0.26]	N/A	N/A	N/A	N/A	N/A
	C-index	0.61 [0.59, 0.64]	N/A	N/A	N/A	N/A	N/A

Values are presented as metric [95% confidence interval]. P-values were calculated using two-sample bootstrap tests. N/A (not applicable) denotes instances where the metric could not be robustly estimated via bootstrapping, typically due to an insufficient number of events within the subgroup.

Abbreviations: C-index, concordance index; IAUC, integrated area under the time-dependent receiver operating characteristic curve; IBS, integrated Brier score.

Supplementary Table 7. Subgroup analysis based on IDH mutation across validation cohorts.

Center	Metric	(1) Wildtype	(2) Mutant	P value (1) vs (2)
**Internal Validation**	IAUC	0.80 [0.40, 0.61]	N/A	N/A
	IBS	0.14 [0.16, 0.23]	0.03 [0.01, 0.08]	<0.001
	C-index	0.71 [0.63, 0.75]	0.79 [0.57, 0.94]	0.342
**External Validation 1**	IAUC	0.62 [0.35, 0.59]	N/A	N/A
	IBS	0.21 [0.21, 0.28]	N/A	N/A
	C-index	0.58 [0.48, 0.66]	N/A	N/A
**External Validation 2**	IAUC	0.68 [0.41, 0.54]	0.87 [0.29, 0.57]	0.556
	IBS	0.17 [0.19, 0.22]	0.03 [0.02, 0.08]	<0.001
	C-index	0.62 [0.59, 0.67]	0.77 [0.67, 0.90]	0.032
**External Validation 3**	IAUC	0.68 [0.46, 0.55]	N/A	N/A
	IBS	0.21 [0.24, 0.26]	N/A	N/A
	C-index	0.61 [0.59, 0.64]	N/A	N/A

Values are presented as metric [95% confidence interval]. P-values were calculated using two-sample bootstrap tests. N/A (not applicable) denotes instances where the metric could not be robustly estimated via bootstrapping, typically due to an insufficient number of events within the subgroup.

Abbreviations: C-index, concordance index; IAUC, integrated area under the time-dependent receiver operating characteristic curve; IBS, integrated Brier score.

Supplementary Table 8. Subgroup analysis based on MGMTp methylation across validation cohorts.

Center	Metric	(1) Unmethylated	(2) Methylated	P value (1) vs (2)
**Internal Validation**	IAUC	0.76 [0.33, 0.63]	0.88 [0.23, 0.50]	0.282
	IBS	0.14 [0.16, 0.25]	0.07 [0.07, 0.16]	<0.001
	C-index	0.68 [0.58, 0.75]	0.83 [0.75, 0.88]	0.012
**External Validation 1**	IAUC	0.65 [0.29, 0.55]	0.88 [0.22, 0.55]	0.640
	IBS	0.21 [0.23, 0.33]	0.12 [0.20, 0.36]	0.896
	C-index	0.59 [0.48, 0.70]	0.80 [0.72, 0.92]	<0.001
**External Validation 2**	IAUC	0.73 [0.41, 0.64]	0.71 [0.43, 0.58]	0.700
	IBS	0.13 [0.13, 0.19]	0.18 [0.18, 0.23]	0.040
	C-index	0.67 [0.57, 0.74]	0.63 [0.60, 0.70]	0.954
**External Validation 3**	IAUC	0.67 [0.44, 0.63]	0.67 [0.34, 0.52]	0.118
	IBS	0.18 [0.18, 0.23]	0.22 [0.22, 0.29]	0.028
	C-index	0.62 [0.56, 0.67]	0.59 [0.55, 0.67]	0.868

Values are presented as metric [95% confidence interval]. P-values were calculated using two-sample bootstrap tests. N/A (not applicable) denotes instances where the metric could not be robustly estimated via bootstrapping, typically due to an insufficient number of events within the subgroup.

Abbreviations: C-index, concordance index; IAUC, integrated area under the time-dependent receiver operating characteristic curve; IBS, integrated Brier score.

Supplementary Table 9. Subgroup analysis based on Extent of resection across validation cohorts.

Center	Metric	(1) Biopsy	(2) STR	(3) GTR	P value (1) vs (2)	P value (1) vs (3)	P value (2) vs (3)
**Internal Validation**	IAUC	0.72 [0.10, 0.60]	0.85 [0.39, 0.73]	0.87 [0.32, 0.67]	N/A	N/A	N/A
	IBS	0.15 [0.17, 0.36]	0.12 [0.11, 0.22]	0.08 [0.11, 0.18]	N/A	N/A	N/A
	C-index	0.62 [0.42, 0.77]	0.77 [0.65, 0.85]	0.81 [0.75, 0.86]	N/A	N/A	N/A
**External Validation 1**	IAUC	0.71 [0.55, 0.94]	0.65 [0.35, 0.76]	0.75 [0.31, 0.58]	N/A	N/A	N/A
	IBS	0.18 [0.11, 0.25]	0.20 [0.18, 0.30]	0.16 [0.26, 0.38]	N/A	N/A	N/A
	C-index	0.53 [0.22, 0.83]	0.62 [0.43, 0.78]	0.72 [0.62, 0.82]	N/A	N/A	N/A
**External Validation 2**	IAUC	0.95 [0.63, 0.91]	0.87 [0.42, 0.59]	0.69 [0.41, 0.57]	N/A	N/A	N/A
	IBS	0.13 [0.12, 0.25]	0.12 [0.17, 0.23]	0.16 [0.17, 0.22]	N/A	N/A	N/A
	C-index	0.73 [0.61, 0.83]	0.77 [0.74, 0.83]	0.60 [0.57, 0.67]	N/A	N/A	N/A
**External Validation 3**	IAUC	N/A	0.68 [0.43, 0.61]	0.68 [0.43, 0.54]	N/A	N/A	N/A
	IBS	N/A	0.21 [0.22, 0.26]	0.21 [0.23, 0.27]	N/A	N/A	N/A
	C-index	N/A	0.56 [0.53, 0.61]	0.61 [0.58, 0.65]	N/A	N/A	N/A

Values are presented as metric [95% confidence interval]. P-values were calculated using two-sample bootstrap tests. N/A (not applicable) denotes instances where the metric could not be robustly estimated via bootstrapping, typically due to an insufficient number of events within the subgroup.

Abbreviations: C-index, concordance index; IAUC, integrated area under the time-dependent receiver operating characteristic curve; IBS, integrated Brier score.

Supplementary Table 10. Quantitative validation of activation map performance

Center	Accuracy	Sensitivity	Precision	Specificity	AUC	Localization Accuracy
**Internal Validation**	0.979	0.396	0.545	0.989	0.905	0.859
**External Validation 1**	0.981	0.445	0.588	0.989	0.938	0.905
**External Validation 2**	0.976	0.307	0.385	0.984	0.838	0.726
**External Validation 3**	0.985	0.418	0.571	0.992	0.966	0.942

Values represent the mean of each metric. Metrics such as Accuracy, Sensitivity, Precision, Specificity, and AUC are pixel-level evaluations. Localization Accuracy is recorded as 1 if the model-identified significant region overlaps with the ground-truth tumor area, and 0 otherwise. Activation maps are generated using Eigen-Class Activation Mapping (Eigen-CAM).

Supplementary Table 11. Pixel-level AUC stratified by localization accuracy of activation maps

Center	AUC (Accurate Localization)	AUC (Inaccurate Localization)	P value
**Internal Validation**	0.970	0.511	< 0.001
**External Validation 1**	0.990	0.441	0.001
**External Validation 2**	0.965	0.501	< 0.001
**External Validation 3**	0.987	0.631	< 0.001

Pixel-level Area Under the Curve (AUC) stratified by tumor localization accuracy. The table compares model performance across two distinct subsets: 'Accurate Localization,' comprising cases where the tumor region was correctly identified, and 'Inaccurate Localization,' for cases where it was not. Values represent the mean of each metric. Metrics such as Accuracy, Sensitivity, Precision, Specificity, and AUC are pixel-level evaluations. Localization Accuracy is recorded as 1 if the model-identified significant region overlaps with the ground-truth tumor area, and 0 otherwise. Activation maps are generated using Eigen-Class Activation Mapping (Eigen-CAM).

Supplementary Table 12. Feature relevance scores for two representative patients stratified by histopathological subtype: glioblastoma versus oligodendroglioma.

Feature	Patient1			Patient2		
	Attribute	Relevance	SHAP	Attribute	Relevance	SHAP
MRI	-	0.06	-	-	0.05	-
Age	59	-0.00	-	52	0.00	-
Sex	Female	0.01	-	Male	0.00	-
KPS	100	-0.02	0.03	100	-0.01	-0.19
WHO Grade	Grade 4	0.06	0.08	Grade 3	-0.07	-1.34
Histopathology	Glioblastoma	0.07	0.05	Oligodendroglioma	-0.14	-0.26
IDH mutation	Wildtype	0.07	0.03	Mutated	-0.06	-0.64
1p/19q codeletion	Non-codeleted	0.05	0.34	Codeleted	-0.11	-0.11
MGMTp methylation	Unmethylated	0.02	-0.16	Methylated	-0.03	-0.08
Extent of resection	Biopsy	0.07	0.73	Gross Total Resection	0.01	-0.16
Radiotherapy	Received	-0.01	-0.19	Received	-0.01	-0.06
Chemotherapy	Received	-0.02	-0.15	Received	-0.01	0.08
Overall Survival / Survival Status	8.7 months / Death			34.5 months / Death		
Survival Probability1	S(t1=8.7) = 57%			S(t1=8.7) = 89%		
Survival Probability2	S(t2=34.5) = 2%			S(t2=34.5) = 52%		

Columns display attribute values and corresponding GlioSurv-derived relevance scores for each patient. Columns display attribute values and corresponding GlioSurv-derived relevance scores and SHAP (SHapley Additive exPlanations) values for each patient. For both scoring methods, positive relevance values indicate features contributing to increased predicted risk, while negative values indicate features contributing to protective effects. Patient 1 (glioblastoma) survived 8.7 months; Patient 2 (oligodendroglioma) survived 34.5 months. Survival probabilities are shown at t1 = 8.7 months and t2 = 34.5 months.

Abbreviations: IDH, isocitrate dehydrogenase; KPS, Karnofsky Performance Status; MGMTp, O6-methylguanine-DNA methyltransferase promoter; MRI, magnetic resonance imaging; WHO, World Health Organization.

Supplementary Table 13. Feature relevance scores for two representative patients stratified by MGMTp methylation status: methylated versus unmethylated.

Feature	Patient1			Patient2		
	Attribute	Relevance	SHAP	Attribute	Relevance	SHAP
MRI	-	0.04	-	-	0.03	-
Age	65	0.16	-	77	0.10	-
Sex	Male	0.00	-	Male	0.00	-
KPS	90	-0.02	-0.00	90	-0.01	0.00
WHO Grade	Grade 4	0.07	0.01	Grade 4	0.04	0.01
Histopathology	Glioblastoma	0.09	-0.00	Glioblastoma	0.05	-0.00
IDH mutation	Wildtype	0.09	-0.02	Wildtype	0.05	0.01
1p/19q codeletion	Non-codeleted	0.06	-0.53	Non-codeleted	0.04	0.31
MGMTp methylation	Methylated	-0.06	-0.31	Unmethylated	0.02	-0.41
Extent of resection	Gross Total Resection	0.01	-0.09	Gross Total Resection	0.01	-0.09
Radiotherapy	Received	-0.01	-0.16	Received	-0.01	-0.17
Chemotherapy	Received	-0.03	-0.17	Received	-0.02	-0.19
Overall Survival / Survival Status	31.6 months / Death			11.3 months / Death		
Survival Probability1	S(t1=31.6) = 51%			S(t1=31.6) = 9%		
Survival Probability2	S(t2=11.3) = 84%			S(t2=11.3) = 54%		

Columns display attribute values and corresponding GlioSurv-derived relevance scores for each patient. Columns display attribute values and corresponding GlioSurv-derived relevance scores and SHAP (SHapley Additive exPlanations) values for each patient. For both scoring methods, positive relevance values indicate features contributing to increased predicted risk, while negative values indicate features contributing to protective effects. Patient 1 (MGMTp methylated) survived 31.6 months; Patient 2 (MGMTp unmethylated) survived 11.3 months. Survival probabilities are shown at t1 = 31.6 months and t2 = 11.3 months.

Abbreviations: IDH, isocitrate dehydrogenase; KPS, Karnofsky Performance Status; MGMTp, O6-methylguanine-DNA methyltransferase promoter; MRI, magnetic resonance imaging; WHO, World Health Organization.

Supplementary Table 14. Feature relevance scores for two representative patients stratified by extent of surgical resection: gross total resection versus subtotal resection.

Feature	Patient1			Patient2		
	Attribute	Relevance	SHAP	Attribute	Relevance	SHAP
MRI	-	0.01	-	-	0.03	-
Age	66	0.17	-	69	0.09	-
Sex	Male	0.00	-	Male	0.00	-
KPS	90	-0.02	-0.00	100	-0.01	-0.03
WHO Grade	Grade 4	0.07	0.01	Grade 4	0.04	0.00
Histopathology	Glioblastoma	0.09	0.04	Glioblastoma	0.05	0.03
IDH mutation	Wildtype	0.09	0.01	Wildtype	0.05	0.01
1p/19q codeletion	Non-codeleted	0.06	0.12	Non-codeleted	0.03	0.32
MGMTp methylation	Unmethylated	0.03	-0.37	Unmethylated	0.02	-0.40
Extent of resection	Gross Total Resection	0.01	-0.12	Subtotal Resection	0.00	-0.00
Radiotherapy	Received	-0.01	-0.17	Received	-0.01	-0.22
Chemotherapy	Received	-0.03	-0.20	Received	-0.01	-0.22
Overall Survival / Survival Status	22.3 months / Death			9.8 months / Death		
Survival Probability1	S(t1=22.3) = 59%			S(t1=22.3) = 21%		
Survival Probability2	S(t2=9.8) = 88%			S(t2=9.8) = 58%		

Columns display attribute values and corresponding GlioSurv-derived relevance scores for each patient. Columns display attribute values and corresponding GlioSurv-derived relevance scores and SHAP (SHapley Additive exPlanations) values for each patient. For both scoring methods, positive relevance values indicate features contributing to increased predicted risk, while negative values indicate features contributing to protective effects. Patient 1 (gross total resection) survived 22.3 months; Patient 2 (subtotal resection) survived 9.8 months. Survival probabilities are shown at t1 = 22.. months and t2 = 9.8 months.

Abbreviations: IDH, isocitrate dehydrogenase; KPS, Karnofsky Performance Status; MGMTp, O6-methylguanine-DNA methyltransferase promoter; MRI, magnetic resonance imaging; WHO, World Health Organization.

Supplementary Table 15. Missing Data Proportions for Each Variable Across Cohorts

Characteristics	Internal Institutional set (n=891)	External Validation sets		
		External Institutional set (n=84)	UCSF (n=470)	UPenn (n=499)
**Age**	0.0%	0.0%	0.0%	0.0%
**Sex**	0.0%	0.0%	0.0%	0.0%
**Karnofsky performance status**	7.6%	0.0%	100.0%	85.0%
**WHO Grade**	0.0%	0.0%	0.0%	0.0%
**Histopathology**	0.0%	0.0%	0.0%	0.0%
**IDH mutation**	0.0%	0.0%	0.0%	0.0%
**1p/19q co-deletion**	3.4%	0.0%	19.4%	100.0%
**MGMTp methylation**	1.8%	0.0%	16.8%	48.9%
**Extent of resection**	0.8%	0.0%	0.0%	4.8%
**Radiation therapy**	0.0%	100.0%	100.0%	100.0%
**Chemotherapy**	0.0%	100.0%	100.0%	100.0%

Values are presented as the percentage of missing data.

Abbreviations: IDH, isocitrate dehydrogenase; MGMTp, O6-methylguanine-DNA methyltransferase promoter; UCSF, University of California, San Francisco; UPenn, University of Pennsylvania; WHO, World Health Organization.

Supplementary Table 16. Performance comparison of the proposed masked cross-attention with baseline imputation methods across validation cohorts.

Center	Metric	(1) Masked Cross-Attention	(2) Mode	(3) MICE	P value (1) vs (2)	P value (1) vs (3)
**Internal Validation**	IAUC	0.86 [0.82, 0.91]	0.84 [0.79, 0.89]	0.78 [0.73, 0.85]	<0.001	<0.001
	IBS	0.10 [0.08, 0.12]	0.10 [0.09, 0.12]	0.13 [0.11, 0.15]	0.002	<0.001
	C-index	0.80 [0.76, 0.85]	0.80 [0.75, 0.84]	0.75 [0.70, 0.80]	0.422	0.069
**External Validation 1**	IAUC	0.75 [0.64, 0.83]	0.74 [0.66, 0.83]	0.69 [0.60, 0.79]	0.821	<0.001
	IBS	0.17 [0.14, 0.20]	0.19 [0.15, 0.22]	0.18 [0.16, 0.21]	<0.001	<0.001
	C-index	0.70 [0.62, 0.78]	0.69 [0.61, 0.76]	0.65 [0.56, 0.73]	0.406	0.167
**External Validation 2**	IAUC	0.78 [0.74, 0.82]	0.77 [0.73, 0.81]	0.76 [0.72, 0.78]	<0.001	<0.001
	IBS	0.14 [0.13, 0.16]	0.18 [0.16, 0.20]	0.15 [0.13, 0.16]	<0.001	<0.001
	C-index	0.71 [0.67, 0.74]	0.71 [0.68, 0.75]	0.70 [0.68, 0.73]	0.571	0.998
**External Validation 3**	IAUC	0.68 [0.64, 0.72]	0.65 [0.61, 0.69]	0.62 [0.58, 0.66]	<0.001	<0.001
	IBS	0.21 [0.20, 0.22]	0.29 [0.28, 0.31]	0.25 [0.24, 0.26]	<0.001	<0.001
	C-index	0.61 [0.59, 0.64]	0.61 [0.59, 0.64]	0.58 [0.55, 0.60]	0.430	0.037

This table compares the downstream performance of our proposed masked cross-attention against two strong baseline methods: mode imputation and multivariate imputation by chained equations (MICE). Values are presented as metric [95% confidence interval]. P-values were calculated using two-sample bootstrap tests. N/A (not applicable) denotes instances where the metric could not be robustly estimated.

Abbreviations: C-index, concordance index; IAUC, integrated area under the time-dependent receiver operating characteristic curve; IBS, integrated Brier score; MICE, multivariate imputation by chained equations.
